# Impact of host age on viral and bacterial communities in a waterbird population

**DOI:** 10.1038/s41396-022-01334-4

**Published:** 2022-11-01

**Authors:** Sarah C. Hill, Sarah François, Julien Thézé, Adrian L. Smith, Peter Simmonds, Christopher M. Perrins, Lia van der Hoek, Oliver G. Pybus

**Affiliations:** 1 Department of Pathobiology and Population Sciences, Royal Veterinary College, London, UK; 2 Department of Biology, University of Oxford, Oxford, UK; 3 UMR EPIA, Université Clermont Auvergne, INRAE, VetAgro Sup, Saint-Genès-Champanelle, France; 4 Nuffield Department of Medicine, University of Oxford, Peter Medawar Building, South Parks Road, Oxford, UK; 5 Amsterdam Institute for Infection and Immunity, Amsterdam, The Netherlands; 6 Laboratory of Experimental Virology, Department of Medical Microbiology and Infection Prevention, Amsterdam UMC location University of Amsterdam, Amsterdam, The Netherlands

**Keywords:** Metagenomics, Molecular biology, Microbial ecology, Next-generation sequencing, Infectious diseases

## Abstract

Wildlife harbour pathogens that can harm human or livestock health and are the source of most emerging infectious diseases. It is rarely considered how changes in wildlife population age-structures or how age-stratified behaviours might alter the level of pathogen detection within a species, or risk of spillover to other species. Micro-organisms that occur in healthy animals can be an important model for understanding and predicting the dynamics of pathogens of greater health concern, which are hard to study in wild populations due to their relative rarity. We therefore used a metagenomic approach to jointly characterise viral and prokaryotic carriage in faeces collected from a healthy wild bird population (*Cygnus olor*; mute swan) that has been subject to long-term study. Using 223 samples from known individuals allowed us to compare differences in prokaryotic and eukaryotic viral carriage between adults and juveniles at an unprecedented level of detail. We discovered and characterised 77 novel virus species, of which 21% belong putatively to bird-infecting families, and described the core prokaryotic microbiome of *C. olor*. Whilst no difference in microbiota diversity was observed between juveniles and adult individuals, 50% (4/8) of bird-infecting virus families (picornaviruses, astroviruses, adenoviruses and bornaviruses) and 3.4% (9/267) of prokaryotic families (including *Helicobacteraceae*, *Spirochaetaceae* and *Flavobacteriaceae* families) were differentially abundant and/or prevalent between juveniles and adults. This indicates that perturbations that affect population age-structures of wildlife could alter circulation dynamics and spillover risk of microbes, potentially including pathogens.

## Introduction

Wildlife harbour a range of pathogens that can threaten human and livestock health, and are the source of most emerging infectious diseases in humans [1]. The risk of cross-species emergence depends on the frequency and nature of contacts between reservoir and recipient species (e.g., [2, 3]). Previous studies have explored how these risks are affected by extrinsic ecological and environmental factors, such as habitat disruption, fragmentation, or changes in animal movement caused by climate and land-use change [4–9]. In contrast, the contributions of intrinsic demographic factors to infectious disease emergence are poorly understood, notably of host age-structure. Yet if the diversity and abundance of pathogens in a source population vary by age, environmental disturbances that are age-specific or change host age-structure (e.g., [10–14]) could modulate the risk of disease spillover. For example, zoonotic potential will be amplified if age-classes with high pathogen prevalence or load more frequently exhibit behaviours that increase inter-specific contact rates, such as altered home range size [15, 16], seasonal migration [17, 18], foraging patterns [19], or presence in different environmental niches [20, 21]. A better understanding of how age contributes to eukaryotic viral and prokaryotic carriage in wild animal populations is therefore critical for predicting infectious disease spread and spillover risk. Such knowledge would contribute also to conservation by informing how age-structured dispersal might modulate the risk of disease transmission among fragmented populations of endangered species [6].

Although it is well known that age affects the dynamics and prevalence of many diseases, wildlife age structures have been investigated in the context of infection dynamics only for a limited number of pathogens (e.g., [22–24]). In part, this is because pathogenic micro-organisms are challenging to study in the wild due to a low chance of detecting them opportunistically in sufficiently well-characterised wild populations. Non-pathogenic micro-organisms could be an important model for understanding and predicting the epidemiology of micro-organisms of greater health concern.

Carefully structured metagenomic studies of well-characterised, healthy wild animal populations could be used to understand the demographic factors that shape microbial diversity and distribution. However, metagenomic studies that consider the impact of different ecological drivers on microbial community composition typically focus on comparing microbial diversity across species or populations (e.g., [25]), rather than among individuals within a species [26]. Most metagenomic studies that capture individual information on age and sex focus solely on bacterial diversity (e.g., [27–29]) and do not characterise viruses, which have higher zoonotic emergence potential [30]. To our knowledge, no studies have jointly investigated eukaryotic viral and prokaryotic diversity varies within the same wild individuals [26]. This makes it difficult to directly compare the relative impact of population age-structures on different types of infectious agent. One previous study of the Ruddy turnstone (*Arenaria interpres*) virome has indicated that avian age may influence RNA-virus community structure, but DNA viruses were excluded and inferences were limited by the use of only five pooled samples [31]. Negrey et al (2020) showed that older adult male chimpanzees had higher viral richness than younger adult chimpanzees [32]. However, due to their focus on senescence, they did not consider samples from juveniles who we predict might have highest levels of viral diversity due to immune naivety [33].

Here we investigate how the demographic structure of a wild animal population influences the prevalence and diversity of both prokaryotes and eukaryotic viruses (i.e., excluding bacteriophages) within it. To achieve this, we undertook a long-term, age-structured survey of a large, healthy colony of free-living mute swans (*Cygnus olor*), a long-lived waterbird species commonly found in Europe. We conducted a large-scale non-invasive metagenomic study to comprehensively characterise and analyse the eukaryotic viral and prokaryotic communities in this population, resulting in an exceptionally large metagenomic data set comprising 6.4 billion sequence reads generated from 223 samples collected across an entire year of observation. These genomic data were combined with a rich database of demographic data for individually identified birds. This allowed us to investigate the differential impact of host age structure on microbial carriage with unprecedented detail in a natural population.

## Material and methods

### Study population

**Fig. 1:**
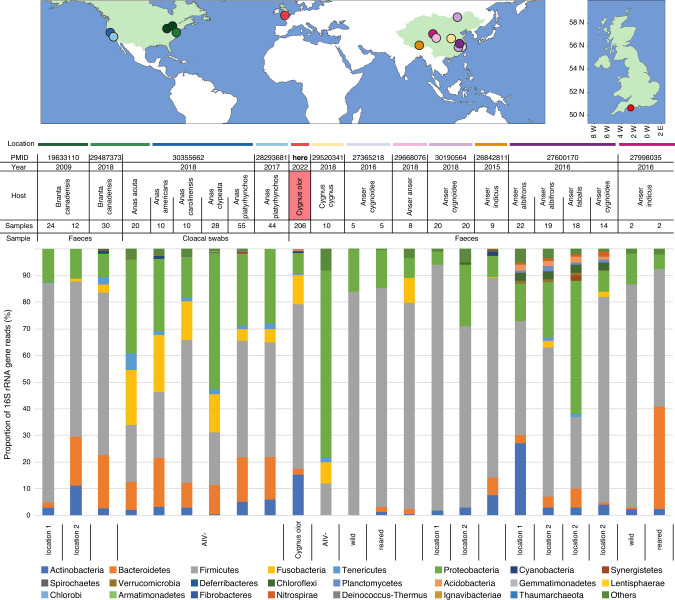
Comparison of the composition of faecal prokaryotic communities of *C. olor*, at the phylum level, to the results obtained by other metagenomic studies conducted on wild *Anatidae* species. The bibliographic search was conducted of PubMed on 16 September 2019 using the Adjutant package in R, using the search ‘(bird OR avian) AND (bact metagenomic OR 16S OR bact microbio)’ (1424 papers). We removed papers (i) that were not primary research articles, (ii) whose samples were not taken from living birds, (iii) that targeted one or few specific prokaryote taxa, and (iv) that did not target wild populations of *Anatidae* species. We consequently retained a total of 11 papers on *Anatidae* prokaryotic microbiomes (corresponding PMID are provided on top of the figure). Proportion of 16S rRNA gene reads at the phylum level was obtained by screening the results of these 11 published studies. The upper maps show the approximate location of sampling of each of these studies as indicated by corresponding coloured dots and horizontal bars.

Our study population is located at the Fleet Lagoon (Dorset, UK; (50.65378^◦^N), 2.60288^◦^W) (Figs. 1, Fig. S1). The population is a semi-habituated colony of wild, free-living mute swans (*Cygnus olor*) that has been subject to long-term (50 years) ecological and ornithological study. The colony comprises approximately 600 to 1000 birds (Fig. S2) and is unusually large for this species. Population numbers are often higher in the early summer following hatching of cygnets, and in the mid-summer as birds immigrate from local areas into the lagoon for moulting. The swans are ringed and detailed information (age, sex, weight, mating data, parentage) is available for most birds [34, 35]. Supplementary food (wheat grain) is provided from spring to autumn, and more rarely in winter. Families with young cygnets are usually also provided with supplementary grass cut from around the site between May and October, when the cygnets are <4 months old. A fence around the site reduces terrestrial predation. Survival rates of first-year and adult birds are similar to those elsewhere [36, 37], but the survival rates of birds in their second and third years are slightly higher in the studied colony [34]. Overall longevity is approximately 9 years for birds that survive their first two years of life, and is therefore similar to other populations in Europe [37, 38]. There are biannual attempts to catch and survey the whole colony, and the birds are vaccinated against duck virus enteritis.

For the purposes of statistical analyses, birds were considered as juveniles when <3 years old (calendar year of sampling minus the calendar year of hatching), and otherwise considered as adults. This grouping follows biological definitions of juvenile vs adult mute swans, as mute swans often begin to breed in their third year [39]. Further, age-structured mortality rates in our population are higher in the first two years of life than during most of adult life [37].

### Sampling

Faecal samples were collected non-invasively on 8 occasions between April 2016 and June 2017, with each visit lasting 2-3 days. Samples could not be collected between December 2016 and February 2017 because of a highly pathogenic avian influenza virus subtype H5N8 outbreak that killed approximately 37% of <1 year old birds and 5% of older birds [40]. Birds were observed when on land near the lagoon, and samples were taken at random from ringed swans that defecated. Typically, samples were collected within 5 min, although sometimes samples were collected from sedentary birds that had clearly recently defecated whilst the researcher patrolled the habituated flock. For the latter case, samples were collected within approximately 5–30 min. Each swan’s plastic leg ring (5 cm long and engraved with a unique three letter code) is large enough to be observed without disturbing the bird (Fig. S1c). Approximately 0.5 ml of faecal material was collected from the top surface of the faeces to avoid environmental contamination with a single-use sterile plastic spatula (Corning Ltd, Corning, New York) into a 1.5 ml sample tube containing 1.0 ml of Universal Transport Media (COPAN Diagnostics, Murrieta, California). Each tube was shaken vigorously and kept on ice for up to an hour, before being placed at -80^◦^C in a freezer situated at the field site. All subsequent transport was conducted on dry ice. None of the birds studied here are known to have died within 7 weeks following sampling, and none had apparent symptoms of disease at the time of sampling.

### Sample preparation and sequencing

Samples were selected from a large set of opportunistically-collected material to achieve a sample set that was relatively balanced by sex, year of hatching and sampling date. Faecal samples were processed using previously published methods [41]. Briefly, enrichment for encapsidated viral nucleic acids relative to eukaryotic cells and bacteria was conducted by centrifugation and selection of supernatant. Filtration was not performed so that any large viruses and bacterial data could be also retained. Residual DNA was digested with DNase I [41]. The Boom method was used for extraction of DNA and RNA nucleic acids [42]. Reverse transcription was performed using non-ribosomal random hexamers [43]. Second strand DNA synthesis was performed with Klenow fragment DNA polymerase. Nucleic acids were purified by phenol/chloroform extraction and ethanol precipitation [41]. The dsDNA concentrations were measured using a Qubit high sensitivity dsDNA kit on a Qubit 3.0 fluorimeter (Thermofisher Scientific, Waltham, Massachusetts). Samples were barcoded and paired-end library preparation was performed for all samples using a Nextera XT kit (Illumina Inc, San Diego, California). The samples were sequenced in three multiplexes on an HiSeq 4000 (Illumina) to generate 150 bp paired end reads. Specifically, the first batch was run using a multiplex of 10 samples on a single lane to test the protocol. A second batch of 117 swan samples and 1 non-swan control sample was sequenced across 5 lanes and a third batch of 96 samples and 2 non-swan control samples was sequenced across 4 lanes.

In the first library, a blank swab placed in transport media at the field site was prepared to the point of dsDNA alongside other samples. The presence of dsDNA was undetectable when tested on a Qubit 3.0 fluorometer, but the sample was not sequenced. In the second library, we included a “non-swan” control sample (red junglefowl semen sample) that was prepared and sequenced alongside the swan samples. In the third library, we also included two “non-swan” rodent faecal samples, which were prepared separately but sequenced on the same Illumina run. Samples were processed agnostically of avian age and were not sorted by age.

### Viral genome reconstruction

Sequencing adaptors were removed and reads were filtered for quality (q30 quality and read length >45nt) using cutadapt 1.18 [44]. Cleaned reads were assembled *de novo* into contigs using SPAdes 3.12.0 (k-mer lengths 21, 33, 55, 77) [45]. Taxonomic assignment was achieved on contigs of length >900 nt through searches against the NCBI RefSeq viral database (downloaded in July 2019) using DIAMOND 0.9.22 with an e-value cutoff of <10^-5^ [46]. All contigs that matched eukaryotic virus sequences were selected and used as queries to perform reciprocal searches against the NCBI non-redundant protein sequence database with an e-value cut-off of <10^-3^ in order to eliminate likely false positives [47]. Virus sequences were classified into viral operational taxonomic units (vOTU – equivalent to contigs). vOTUs were used rather than species because many of the assembled genomes belong to novel and currently unclassified taxa. vOTU contig completion and coverage was assessed by iterative mapping using BOWTIE2 2.3.4.3 [48]. Putative open reading frames (ORFs) were identified using ORF finder (length cutoff >300 nt) on Geneious Prime 2019.1.1 [49]. All subsequent analyses focused only on full or near-full vOTU coding sequences (based on alignment of those genomes with their closest relatives), thereby discarding vOTUs with partial CDS (<90% of CDS).

### Virus discovery and taxonomic assignment

To infer if vOTUs belonged to novel species, their full-length genomic sequences or the predicted sequence of full-length viral proteins were compared with the genomes of the ten most similar viruses, as identified using the similarity searches described above. Each vOTU was aligned using MAFFT v7.388 [50, 51] or MUSCLE 3.8.425 (16 iterations) [52] using default settings. Alignments were visualised using SDT 1.2 [53] and genomes were classified as belonging to already described or novel species according to the species demarcation thresholds recommended by the ICTV (https://talk.ictvonline.org/).

### Phylogenetic analyses

Phylogenetic trees were estimated using maximum likelihood methods for all vOTUs that might represent novel species, in order to place them within currently known viral diversity and to explore their host range. Representative sets of polymerase- or capsid-coding proteins were extracted from the NCBI non-redundant database for each taxonomic group in which the vOTUs were classified. For segmented viruses such as the *Nodaviridae*, in which genes for these proteins occur on different segments, we used both protein sequences.

Amino acid sequences were aligned using MUSCLE 3.8.425 (16 iterations) with default settings [52] or MAFFT v7.388 with the L-INS-i algorithm [50, 51]. Genomic sequences downloaded from NCBI that could not be reliably aligned with the sequences generated here due to high amino acid divergence were removed and the dataset subsequently realigned. No reference sequences were removed during this process. All alignments were trimmed manually to focus on more conserved genome regions and to remove regions that could not be reliably aligned. Amino acid substitution model testing was performed using ProtTest 3.4.2 [54]. Phylogenetic trees were constructed in RAxML 8.2.11 [55] using 1000 bootstrapped replicates. Trees were mid-point rooted and visualized with FigTree 1.4 (http://tree.bio.ed.ac.uk/software%20/figtree/). Phylogenetic placement was used to determine whether the putative host of each detected virus species was likely a vertebrate, or an invertebrate or plant present in the swans’ diet/environment.

### Compositional analysis to support host assignments of newly discovered eukaryotic viruses

To independently corroborate the assigned host taxa for the picornaviruses and related ssRNA viruses detected in the study, discriminant analysis using virus composition (4 genomic mono-nucleotide frequencies and 16 dinucleotide observed / expected representations, generating 20 total parameters) was conducted using parameters based on 277 representative full genome sequences from the order *Picornavirales* and for caliciviruses (sequences from the ICTV Virus Metadata Resource (VMR), MSL36; https://talk.ictvonline.org/taxonomy/vmr/). This method has been previously shown to be effective at discriminating between host type (plants, vertebrates, invertebrates) [56]. Compositional analyses were 94.6% accurate at assigning vertebrate, invertebrate and plant hosts in the VMR control dataset (Table S1).

### Prokaryotic taxonomic assignment

The taxonomic assignment of 16S rRNA gene bacterial and archaeal reads was assessed up to the genera scale using kraken 2.0.7 [57] and bracken 2.2.0 [58], on the RDP v11.5 database [59] with a k-mer length of 35nt.

### Sensitivity test of high-throughput sequencing data

Sequence read data was used to evaluate if bird age affects the differential abundance of viral and microbial taxa. The analysis assumed that sequence read counts were representative of the amount of viral nucleic acid contained in faecal samples. To investigate whether this assumption was likely reasonable we designed two qPCRs against two commonly detected virus species in our study population: one species of genus *Avastrovirus* (*Astroviridae*) and common lineages detected from one species of *Megrivirus* (*Picornaviridae*). cDNA was made using the Protoscript II First Strand cDNA Synthesis Kit (New England Biolabs) and random hexamer primers (Bioline) [43]. qPCRs were set up using the PowerUp SYBR Green Master Mix kit (Applied Biosystems) according to the manufacturer’s instructions, using 5μl of cDNA in a 20μl reaction and 0.5μM of each primer. Primers and annealing temperatures are given in Table S2. Technical replicates were run for all samples in triplicate. A 1:5 dilution series made from the sample with the highest read count for each virus was used in triplicate as a standard curve, with 6 points for the avastrovirus assay and 7 points for megrivirus assay (shown in Fig. S3). Ct values generated from qPCRs were compared with the number of reads per million total reads of different virus species using linear models.

### Statistical analyses

First, data representing the number of reads per prokaryotic or viral taxa per sample were standardised to allow inter-sample comparisons. All statistical analysis was performed using R v 3.6.1 and RStudio 1.2.5019 software [60, 61]. Samples were discarded if they were outliers in term of the completeness of their prokaryotic rarefaction curves at the family scale: samples whose rarefaction curves did not asymptote were removed. Discarding of outliers based on viral rarefaction curves was not undertaken because most viral rarefaction curves did not plateau. Next, we limited taxonomic binning artefacts and potential inter-sample contamination by applying an abundance threshold of >1/1,000,000 reads/sample for prokaryotes and >1/10,000,000 reads/sample for viruses. To enable comparison between viral taxa that have different genome lengths, the number of virus reads was divided by the length of the viral contig to which it mapped (kb).

We conducted assessments of abundance (in terms of read counts) of both prokaryotes and viruses, and assessments of prevalence and diversity for prokaryotes only because viral rarefaction curves did not asymptote. In analyses of viruses, we focus on only coding regions (CDS) that could be assembled completely, or already characterised viral taxa.

The impact of bird age on prokaryotic community α-diversity was evaluated using the Shannon and Simpson diversity indices, and on β-diversity using Bray-Curtis dissimilarity index. The effect of bird age, sex, and seasonality on the composition of prokaryotic communities was also determined by one-factor PERMANOVAs with 10,000 permutations on Bray–Curtis matrix, using the adonis function of the VEGAN package [62]. Permutational tests of dispersions (PERDISPs) using the function permutest.betadisper (999 permutations, pairwise) were performed to assess whether significant effects could be influenced by differences in group dispersion [63]. Statistical significance of PERMANOVA results was assumed when *p* < 0.05 after application of a Bonferroni correction.

The effects of host age on microbial abundance were assessed using Wilcoxon or Kruskal-Wallis tests. Tests were conducted at the family and genus levels for prokaryotes, and at the family and species levels for viruses. Samples containing no putative bird-infecting vOTUs were removed. We investigated and compared seven normalisation methods for inter-sample correction: non-normalised data, counts per million (cpm), upper quartile (q75), trimming mean of M-values (TMM), Relative Log expression (RLE), min-max, and Cumulative Sum Scaling (CSS). As there was little impact of the normalisation method on the results we obtained, we considered results supported by more than three of the seven aforementioned methods to be statistically significant (after adjustment for multiple testing).

The impact of bird age on prokaryote prevalence was tested using Fisher’s exact tests. Rare taxonomic groups (occurring in <5 samples) were not considered for abundance and prevalence analyses. Multiple comparison test adjustment of *p* values was performed using the Benjamini-Hochberg (BH) method [64] for prokaryotes and the Benjamini-Yekutieli (BY) method [65] for viruses. We found high correlation coefficients among the abundances of some prokaryotic taxa but not among those for viruses, and therefore used the BH method for prokaryotes to accommodate this correlation (the BH method is most appropriate when there is dependence in abundance or prevalence among taxa, whereas the BY method is appropriate for independent taxa). We excluded zero counts when calculating odds ratios and their corresponding 95% confidence intervals. For any taxa determined to be differentially abundant or prevalent by age, Kruskal-Wallis tests with BY *p* value correction were used to determine whether the season of sampling impacted the abundance or prevalence of taxa. Sampling dates were aggregated into seasons, defined as: pre-hatching reproduction season (March 2017, early May 2016), hatching season (June 2016), moulting season (July and August 2016), and migration (October to November 2016).

## Results

### Population-level assessment of microbial community

We sequenced the prokaryotic and eukaryotic viral communities from 223 individually identifiable and known-age mute swan samples collected during one year of sampling at the Fleet Lagoon, UK. A summary of the 223 collected samples and of their associated metadata (age, sex, sample collection date) is presented in Table S3 and Fig. S1. After sequencing a total of 6,447,121,538 reads were obtained. After filtering for quality, a total of 6,095,650,474 cleaned reads were assembled *de novo* into contigs. Following data cleaning 205 samples yielded data of sufficient quality for analysis, each of which yielded between 3,873,397 and 46,437,718 reads (average of 29,015,008 reads). Of these, 193 samples contained putative bird-infecting vOTUs. On average 43.9% (±9.2% SD) of reads represent prokaryotic 16S rRNA gene sequences whilst only 0.5% (±3.0% SD—median: 0.016%) of reads represent eukaryotic viruses. Rarefaction curves showed that the full prokaryotic richness of each faecal sample was recovered in each bird at the family and genus scales.

To investigate the presence of contamination, we mapped reads from the non-swan samples against the genomes of identified putative swan-infecting viruses. None of the 352,000,000 rodent samples mapped against putative swan-infecting viruses. Twenty four of 6,050,346 reads in the red junglefowl sample mapped to a genome of a virus from the species *Megrivirus A* (the most abundant virus amongst the swan samples).

**Fig. 2:**
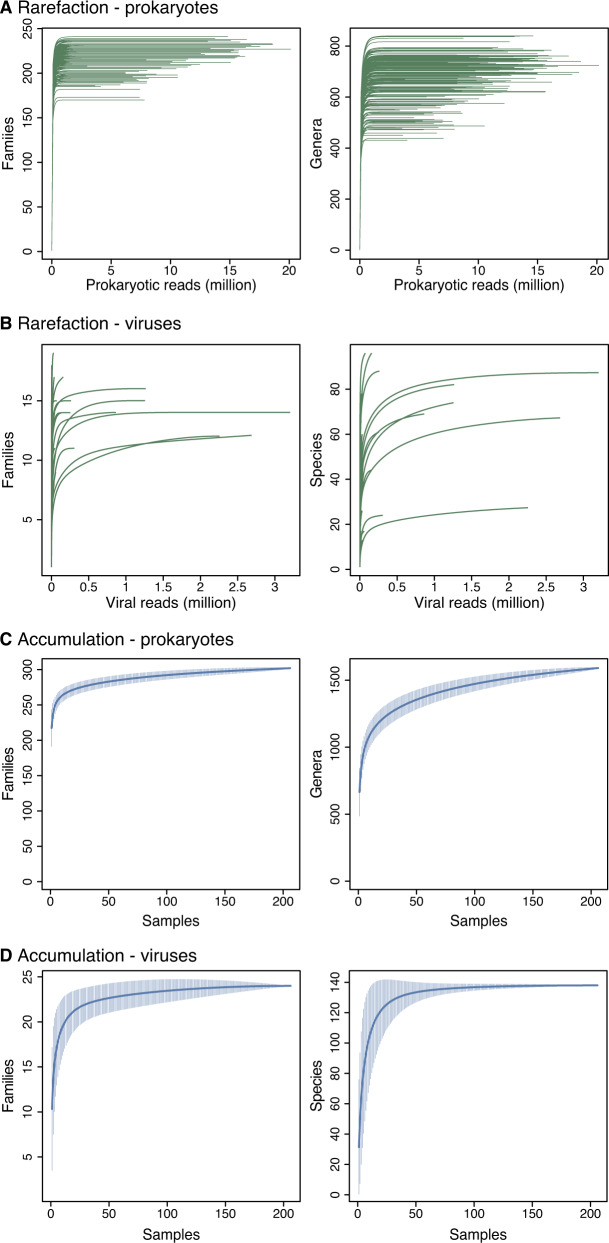
Microbial rarefaction and accumulation curves. Rarefaction curves of (**A**) prokaryotic communities at the family and genus levels; (**B**) viral communities at the family and species levels. Accumulation curves of (**C**) prokaryotic communities at the family and genus levels; (**D**) viral communities at the family and species levels.

Whilst our primary aim was to assess age-based differences in the gut microbiome, our study is also the first to assess the microbial community of a wild *Anatidae* bird population in Europe (Fig. 1). Accumulation and rarefaction curves suggest that we fully recovered the diversity of prokaryotic families in this *C. olor* population. Specifically, approximately 130 samples were required to recover family level diversity (Fig. 2a, c). The prokaryotic microbiota was diverse but dominated, in terms of 16S rRNA gene read count abundance, by relatively few bacterial taxa (Fig. S4, Table S4). This was confirmed by the high Simpson indices (family median: 0.82; genus median: 0.85) and low Shannon indices (family median: 2.3; genus median: 2.69) (Fig. S5). Across all the sequence data generated, 96% of 16S rRNA gene reads represent only four phyla: the Firmicutes (62%), Actinobacteria (15%), Fusobacteria (11%), and Proteobacteria (8%) (Figs. 1 and Fig. S4a). Further, 95% of reads represent 25 families from 6 phyla (Fig. S4b). The prokaryotic community that we characterised was therefore very similar to those observed in previous metagenomic studies conducted on other *Anatidae* species (Fig. 1), and comparable to those observed in other domesticated and wild avian species [66–68].

We reconstructed 138 eukaryotic viruses contigs corresponding to >90% of the expected full length CDS of each virus based on the whole reference genome of the same virus species or the closest-related species in GenBank. Full contig details, including corresponding GenBank accession numbers, are available in Table S5. The majority of the contigs (73%, 101/138) could be fully classified to the species level under ICTV criteria, and most (76%, 77/101) represent novel species (Table 1, Table S5). The other 37 contigs could not be classified to the species level according to existing ICTV definitions, as either they belonged to segmented virus families for which we could not unambiguously determine associated segments (e.g., *Picobirnaviridae*, *Nodaviridae* and *Partitiviridae* family members), or their closest relatives had not been assigned to existing families (unclassified picornavirales-like viruses).

**Table 1:** Number of previously known and novel species belonging to different viral families.

	Family	Number of species (including *n* novel species)
Bird (*n* = 27)	*Adenoviridae*	1
	*Astroviridae*	2 (2)
	*Birnaviridae*	1
	*Bornaviridae*	1
	*Caliciviridae*	1
	*Circoviridae*	1
	*Coronaviridae*	1
	*Parvoviridae*	8 (6)
	*Picornaviridae*	10 (8)
	*Reoviridae*	1
Diet or environment (*n* = 73)	*Alphaflexiviridae*	1
	*Bromoviridae*	1
	*Circoviridae*	18 (17)
	*Genomoviridae*	1
	*Parvoviridae*	7 (6)
	*Tombusviridae*	6 (6)
	*Totiviridae*	3 (3)
	*Tymoviridae*	2
	Unclassified – *Sobemovirus*	3 (1)
	Unclassified – *Picornavirales*	30 (28)
	*Virgaviridae*	1
Unclear (*n* = 1)	*Genomoviridae*	1
	Total taxa	101 (77)

Viruses are listed by putative host range.

Eukaryotic viral taxa were divided in two categories, “bird-infecting” or “diet/environment-associated”, based on their position in virus phylogenies (see Figs. S6–S20) and available information on virus host range, or the host range of their closest relatives. Accuracy of host-assignment for ssRNA viruses was supported by an independent method of virus genomic nucleotide frequency analysis, which showed 89% concordance with phylogenetically inferred categories (Fig. S21, Table S1). This largely corroborates the host assignments within the limits of accuracy of the composition-based method. We detected a maximum of 5 (minimum = 0, mean = 2) different bird-infecting virus families in each faecal sample, and a maximum of 16 (minimum = 0, mean = 5) different putative bird-infecting virus species in each faecal sample.

Diet/environment-associated viruses (e.g., putative plant- or arthropod-infecting viruses) were more abundant than bird-infecting viruses (79% versus 21% of reads) (Table S6), and we also detected more diet-associated virus species than bird-infecting species (73% of 101 contigs from identified species) (Table 1). Most of the diet-associated species we identified were novel, including 28 picornaviruses and 17 circoviruses. Of the 13 previously described diet-associated species, 7 were plant-infecting viruses already known to be present in Europe (Table 1, Table S7).

The majority of the bird-infecting viruses (*n* = 16/27; 59%), including 8 picornaviruses, 6 parvoviruses and 2 astroviruses, belong to putative novel species (Table 1) [69–71]. We generated complete genomes for these novel species, which doubles the number of viruses described in swans (*Cygnus* spp.). All of the 11 already known species of bird-infecting virus found in our datasets are known to infect *Anatidae* species (Table S7). Four of the 16 bird-infecting virus species that have been identified in swans were present in our samples (Table S8) (*Anseriform dependoparvovirus 1*, *Waterbird 1 orthobornavirus*, and *Cygnus olor circovirus*). We also detected influenza A virus at very low abundance (<10 reads that could not be assembled into a contig).

Taxa that are present in every sample can be considered the common core intestinal microbiota of a population [72]. No bird-infecting virus families were detected in all samples, putatively due to a lower virus genetic material enrichment compared to prokaryotes 16S rRNA gene (Fig. 2). In contrast, we observed that 24 of 48 detected prokaryotic phyla, and 127 of 302 families were present in every sample. All of the core microbiota prokaryotic phyla were bacteria, with the exception of the *Thaumarchaeota* phylum.

### Impact of host age on microbiota richness and composition

We conducted a series of analyses to evaluate the effect of host age structure on the joint prokaryotic microbiome and virome of a wild bird population. First, we tested for differences in faecal prokaryotic microbiota richness and composition between adult and juvenile (<3 years old) birds. About two million reads were sufficient to assess the richness of the prokaryotic communities in each sample (Fig. 2a), whereas the sequencing depth of viruses was insufficient to recover full virus taxon richness (Fig. 2b). We therefore analysed microbial abundance (in terms of read counts) of both prokaryotes and viruses, but prevalence and diversity for prokaryotes only. Our qPCR assays confirmed that reads counts can approximate the relative abundance of viruses in our samples (Fig. S3).

**Fig. 3:**
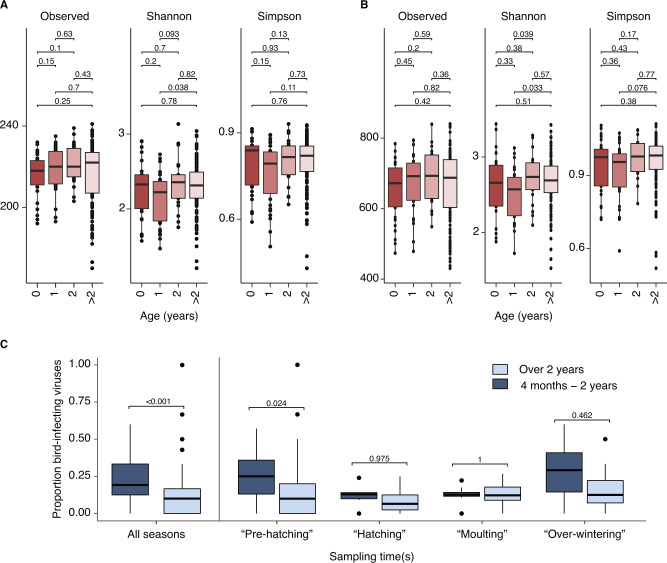
Impact of bird age and seasonality on microbiome composition. Multiple comparisons of the impact of bird age on *C. olor* faecal prokaryotic community diversity at the family scale (**A**) and genus scale (**B**). Diversity analysis comprising observed richness (i.e., the total number of taxa present per sample), Shannon index and Simpson index (y axis). The numbers indicate *p* values. Proportion of viruses that are considered bird-infecting in juveniles compared to adults in the total dataset, and in different seasons. Only those juveniles over 4 months old are shown in **C**.

In general, birds in their first calendar year carried lower numbers of observed prokaryotic taxa than all older birds, and birds aged 1 year of age had lower mean Shannon and Simpson indices than birds of other ages (Fig. 3). However, this effect was not statistically significant (Fig. 3) including when juvenile birds of different ages were grouped in analyses (Fig. S22). PERMANOVAs based on Bray-Curtis dissimilarity matrices show no significant differences in prokaryote community composition between bird age groups after Bonferroni (*p* value = 0.27 at the family level; *p* value = 0.14 at the genus level), although there is a small different in the community composition of the youngest birds (0 years old, *p* value = 0.048 at the family leve; *p* value = 0.042 at the genus level) (Fig. S23, Table S9).

**Fig. 4:**
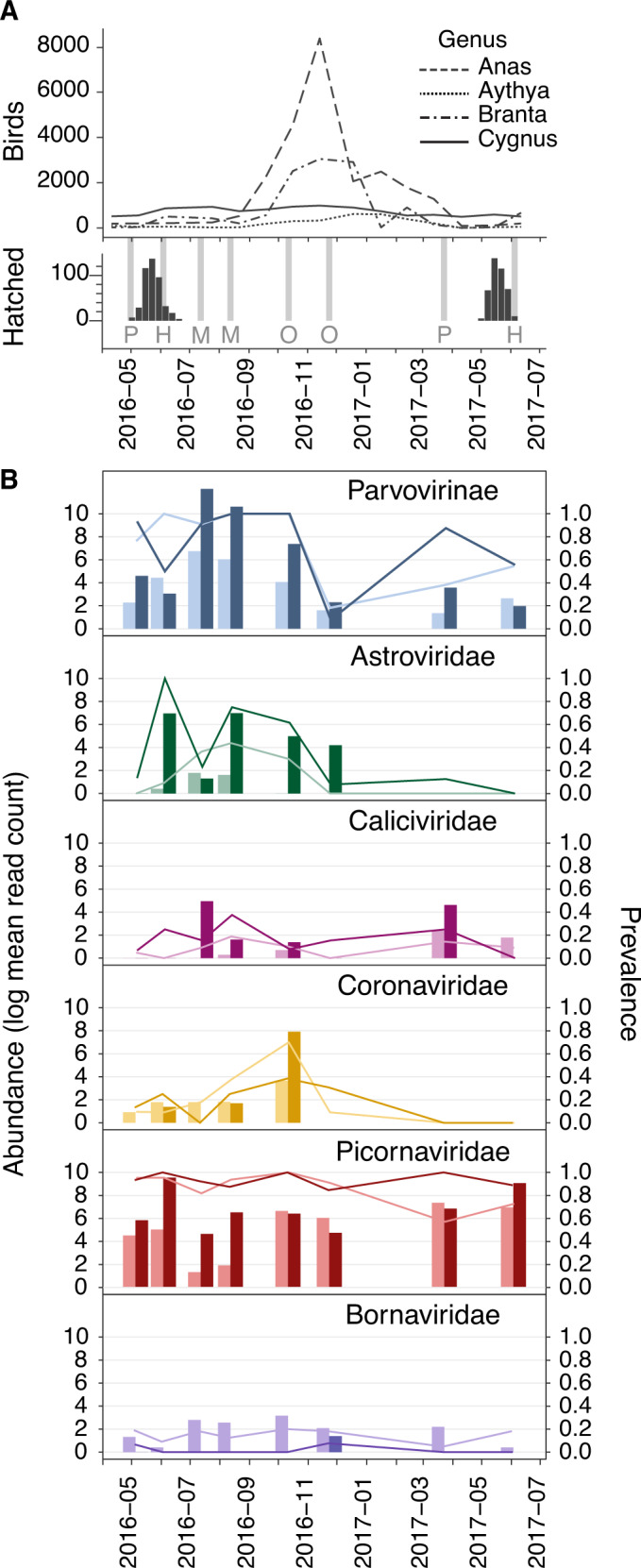
Seasonal changes in viral abundance and prevalence by age. **A** Avian demography over the sampling period. Upper panel: counts of the four most common genera of anseriformes (i.e., ducks, geese, swans) observed on the Fleet Lagoon during the sampling period, according to UK Wetland Bird Survey data (WeBS). Lower panel: number of cygnets hatching per week (black bars). Sampling occasions are indicated by the light grey bars, and seasonal category is marked with a letter; P = pre-hatching reproduction, H = hatching, M = moulting, O = over-winter migration. **B** Abundance (left y-axis, bars) and prevalence (right y-axis, lines) of putative bird-infecting virus groups of *C. olor* faecal microbiota. Abundance is shown as the natural log of the mean number of reads attributed to each taxon when the virus is present (i.e., calculated without considering zero count samples). Darker shades show juveniles, and lighter shades show adults.

Complete viral communities were not recovered for most samples (Fig. 2d) so we could not compare virus community diversity among samples directly. However, we could compare the intra-sample prevalence of bird-infecting and diet-infecting viruses. A higher proportion of the viruses carried by juveniles were categorised as bird-infecting than those from adults (Wilcoxon tests, *p* < 0.001, median difference in virus ratios between juveniles and adults = 0.094 (0.058–0.129)). Very young birds (considered here as <4 months old) have a smaller ranging distance and consume substantially less material than larger adult or older juvenile birds, which may hypothetically expose them to different pathogens. However, our results remained robust when birds <4 months old were removed from analyses (*p* < 0.001, median difference = 0.130 (0.087–0.185)). This trend was present in all sampling seasons, although following *p* value adjustment for multiple testing the difference was only significant during the early spring pre-hatching period (Fig. 3c, see Fig. 4a for seasonal categorisation of sampling events).

**Fig. 5:**
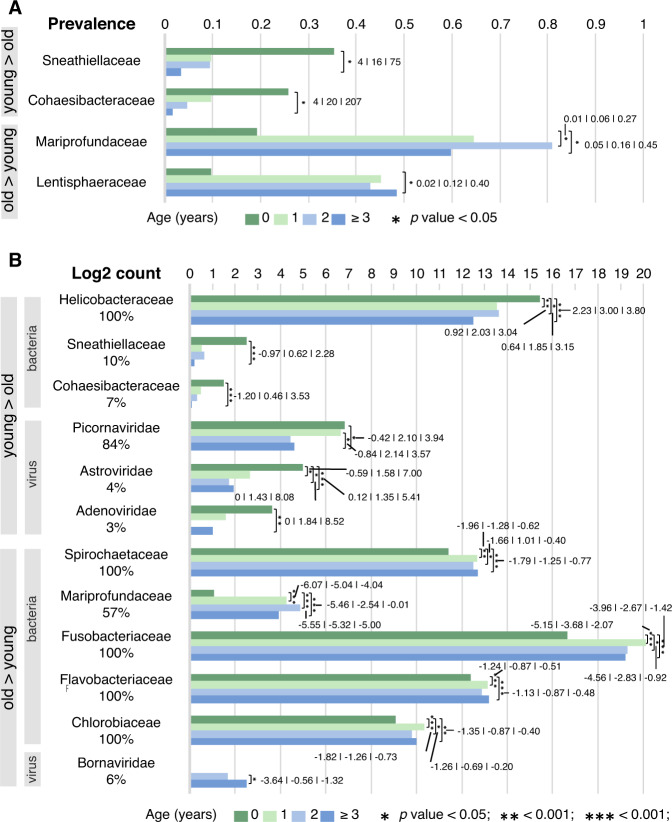
Microbial famillies that differ by abundance or prevalence with bird age. Differently prevalent (**A**) and abundant (**B**) microbial families by age. Only prokaryotes are considered in (**A**), and only those families that are differentially prevalent and/or abundant by age are shown. In (**A**), the horizontal (x) axis indicates prevalence (in percentages). In **B**, the horizontal (x) axis indicates abundance (in log2 count numbers). For both plots, numbers in bold represent the odds ratios, surrounded by 95% lower and upper confidence intervals. Asterisks indicate statistically significant differences. The colours of the bars indicate the age of the birds.

Second, we tested whether specific prokaryotic or viral taxa differed in their abundance or prevalence according to host age. Samples from individual animals contained between 20 and 44 prokaryotic phyla (mean: 39), 170 – 241 prokaryotic families (mean: 218, median: 220) and 431–840 prokaryotic genera (median: 684). We found that a small proportion of prokaryotic families and a higher proportion of bird-infecting virus families, representing 3.4% (9/267) and 50% (4/8) of the tested taxa, respectively, exhibited statistically significant differences in abundance and/or prevalence according to age. Two bacteria families were statistically more prevalent in birds <1 year old compared to adults >2 years old: *Sneathiellaceae* (dominated by *Sneathiella* spp.; 36% versus 3%) and *Cohaesibacteraceae* (dominated by *Cohaesibacter* spp.; 26% versus 2%). These two families were respectively about 16 and 20 times more prevalent (Fig. 5a) and abundant in birds of <1 year old than in adult birds (Fig. 5b, Table S10). Members of the *Helicobacteraceae* family were also much more abundant in young individuals. For example, they were 7.6 times more abundant in <1 year old birds than adults (Fig. 5b). This family was detected in all samples and was dominated by the *Helicobacter* genus.

Similarly, three virus families known to replicate in animal intestines were more abundant in juveniles (particularly in <1 year old birds) than in adults: *Picornaviridae* (4.7-fold more abundant), *Astroviridae* (2.5-fold) and *Adenoviridae* (3.6-fold) (Fig. 5b). Reads belonging to two *Parvovirinae* species (*Mute swan faeces associated chapparvovirus 1* and *Mute swan faeces associated chapparvovirus 3*) were also more abundant in younger than older birds.

In contrast, adults exhibited a higher prevalence and abundance of the *Mariprofundaceae* family (32-fold difference in abundance) and a higher prevalence of *Lentisphaeraceae* (48% prevalence). These two taxa were, respectively, about 6 and 9 times more prevalent in birds over two years than cygnets under one year old (Fig. 5a, b, Table S10). Furthermore, members of the four bacterial families present in all samples (*Spirochaetaceae*, *Flavobacteriaceae*, *Fusobacteriaceae*, and *Chlorobiaceae*) were also more abundant in adults than in juvenile birds (Fig. 5b). Finally, the single species of the *Bornaviridae* family found in this study (*Waterbird 1 orthobornavirus*, detected in 6% of the samples) was absent in birds <2 years old, and was more abundant in adults than in juveniles (Fig. 5b).

Only one taxon that was differentially abundant or prevalent between juveniles and adults was also differentially abundant by season (dates of sampling grouped according to population-level events at the site as shown in Fig. 4a). The *Picornaviridae* family was less abundant during the summer molting period; specifically, it was 4–5 times less abundant than compared to the spring pre-hatching and over-winter periods (*p* value = 0.01). The reduction in abundance of picornavirus reads between early spring and summer does not appear to be directly related to changes in absolute numbers of waterbirds on the Fleet Lagoon, because bird numbers from the four most common *Anatidae* genera on the Fleet Lagoon were relatively stable across that period (Fig. 4a).

*Picornaviridae*, *Parvoviriniae*, *Caliciviridae* and *Bornaviridae* were detected at all sampling occasions (Fig. 4b), whilst *Adenoviridae* and *Birnaviridae* sequences were detected only in few samples (≤3%) from two sampling sessions separated by a short period of time (respectively July—August 2016 and October - November 2016) (Tables S1 and S6). Adenoviruses were more abundant in young birds of <1 year in age, compared to adults (Fig. 5). We therefore speculate that their higher detection rate in July and August 2016 may be driven by high numbers of susceptible juveniles present in the population at that time, following the hatching of 438 cygnets at the site in May and June 2016 (Fig. 4a). Reads attributed to *Coronaviridae* and *Birnaviridae* families were detected mostly during October – November, coinciding with peak immigration of other anseriformes to the Fleet Lagoon (Fig. 4a), perhaps indicating a role for other species in introducing these viruses to the swan population. Nevertheless, we stress that these seasonal changes in abundance were not statistically significant for these taxa, and further longitudinal sampling of this population is required to fully investigate the presence (or absence) of seasonal patterns for the viruses detected here.

## Discussion

Our understanding of wildlife-associated microbial communities is still in its infancy. Here, we concurrently examined the prokaryotic and viral microbiota of a wild animal species, and demonstrated that prevalence and abundance of diverse microbes are associated with the ages of sampled individuals. Strikingly, half (4/8) of the putative bird-infecting virus families studied here were differentially abundant by age. This was far higher than the proportion for prokaryotic taxa, for which only 3.4% (9/267) were differentially prevalent or abundant by age. This highlights the epidemiological and zoonotic importance of considering demographic age structure in addition to absolute host population size when evaluating risk of disease presence, particularly when considering viral diseases.

Almost all viruses that were differently abundant by age, including astroviruses, picornaviruses, adenoviruses and some parvoviruses, were shed in higher loads by juvenile individuals than adults (Fig. 5b). Whilst viruses belonging to two parvovirus species were shed in higher loads by juveniles, abundance of reads from the wider family *Parvovirinae* does not vary significantly with bird age. This may be because of the low abundance of these two parvovirus species compared to the other members of the *Parvovirinae* (Table S6 and Table S10). We hypothesise that the age-based difference in microbial shedding could be due to age-related differences in adaptive immunity to these taxa rather than due to age-related differences in diet. This is consistent with our previous finding that swans in this population maintain long-lasting immune responses to avian influenza virus and that adult swans are consequently less susceptible to influenza-associated mortality [40]. Very young cygnets and nesting adults are more frequently found on freshwater streams leading into the brackish lagoon than non-nesting adults or older juveniles, which tend to forage in the lagoon. However, where tested, our analyses were robust to the exclusion of very young birds <4 months of age. There is no published evidence that juveniles have different diets to older swans. Future research into diets of differently-aged swans would be valuable to exclude an impact of differential exposure to viruses via food choice, or ability of (longer-necked) adult swans to feed at lower water depths.

The only virus that was shed in higher loads by adults than juveniles was waterbird 1 orthobornavirus. Other bornaviruses have been shown to cause persistent infections in birds [73] and mammals [74] that can last for months or years before shedding virus in faeces [75, 76]. We suggest that long persistence of infection explains the higher abundance of waterbird 1 orthobornavirus in adults, although efforts to sequence the virus genome from samples collected longitudinally from the same birds are required to conclusively establish the life cycle of the virus.

The directionality of age-associated differences in abundance was less consistent across bacteria than across viruses. Younger birds were more likely to be carriers of bacteria from the families *Sneathiellaceae* and *Cohaesibacteracea*, and shed higher loads of bacteria from *Sneathiellaceae*, *Cohaesibacteracea* and *Helicobacteriacae*. In contrast, adult birds seemed to harbour a higher prevalence of bacteria belonging to the *Spirochaetaceae* and *Lentisphaeraceae* families, and shed higher loads of bacteria from the *Spirochaetaceae, Mariprofundaceae*, *Fusobacteriaceae*, *Flavobacteriaceae* and *Chlorobiaceae* families. Our findings are likely to be broadly generalisable to other species, because many of the prokaryotic genera detected in our study population are commonly observed at similar frequencies in other species of waterfowl (Fig. 1, Table S10). Bacteria belonging to these families are frequently detected in animals or aquatic environments (Table S10) [77–81].

We did not detect any known zoonotic viruses in this study, although our sampling regime was interrupted (Fig. 4a) by a natural outbreak of highly pathogenic avian influenza virus in December 2016 to February 2017 that preferentially killed younger birds [40]. We hypothesise that the abundance of viruses with zoonotic potential is likely to be similarly affected by host age as the non-zoonotic viruses identified here. This is supported by previous studies; for example, influenza A virus is more prevalent in juvenile than adult birds [82], and seroprevalence against influenza A and flaviviruses such as Usutu virus can be higher in adult birds [33, 83]. The spillover risk of a broad range of viruses, including half of the families of bird-infecting viruses identified here as being differentially abundant according to bird age (i.e. members of *Picornaviridae, Astroviridae*, *Adenoviridae* and *Bornaviridae* families), may be therefore impacted by rapid changes in host demography and age structure. Of note, the *Melegrivirus A* virus species detected is reported to cause hepatic and pancreatic necrosis in turkeys, leading to weight loss, drop in egg production, depression and sudden death [84].

Our study has several limitations. Firstly, whilst supplementary bird-feeding is not unusual in the UK [85, 86], consistent provision of additional food to the studied swans may have impacted microbial communities through altering the microbial species that birds are exposed to, contributing to improved overall bird health, and supporting an unusually large and dense swan population at the Fleet Lagoon. Secondly, we did not use filtration or post-extraction DNase treatments to enrich for virus material because we wanted to capture both the prokaryotic and viral microbiota. Whilst sequencing to high depth allowed us to recover 101 different species of virus and detect how age shaped virus abundance, our data were nevertheless dominated by bacterial reads. Comparatively low abundance of virus reads prevented us from recovering full viral species richness in many samples, and from fully reconstructing some virus genomes due to incomplete coverage. Our choice to focus only on viruses from which whole CDS were recovered means that the dynamics of some viruses, particularly large dsDNA viruses or viruses that typically have very low viral load, were less likely to be captured adequately here. Moreover, some viruses were not considered in this study, specifically (i) those we could not classify at the order, family or genus level, (ii) retroviruses, because of the difficulty in distinguishing between exogenous and endogenous sequences, and (iii) bacteriophages. Thirdly, the first samples were sequenced in 2016 when sequencing of controls was comparatively rare. We therefore did not include consistent negative or any mock community controls. Our study used a manual extraction method, which is expected to introduce lower levels of contamination than robotic plate-based methods during preparation of dsDNA [87]. Minimal detection of putative swan-infecting viruses in control samples from non-swan species indicates that levels of contamination were likely low, but we cannot rigorously assess the level of contamination. Nevertheless, we stress that samples were not sorted by age during processing, so our key results of age-differentiated prokaryotic and viral diversity are unlikely to be severely affected. Finally, our results use stringent tests and correction for multiple testing that may have resulted in low power and false negative results, and we cannot exclude that other microbial taxa may be differentially abundant or prevalent with age than in addition to those reported here.

We conclude that factors that alter demographic structure of a wildlife population, including rapid shifts in breeding-success or age-specific mortality, could have a downstream impact on the dynamics of a wide spectrum of virus and prokaryotic genera. We propose that in order to fully understand the impact of human activities on infection prevalence and risk of infectious-disease spillover from wildlife – particularly from viruses - we first need to understand how human activities impact animal demographic structures, and how microbes are distributed within demographically structured wild populations. This will improve our ability to determine when and where spillover risk from wildlife to domestic animals or human is highest.

## Supplementary information

Supplementary Material

Supplementary Figure 1

Supplementary Figure 2

Supplementary Figure 3

Supplementary Figure 4

Supplementary Figure 5

Supplementary Figure 6

Supplementary Figure 7

Supplementary Figure 8

Supplementary Figure 9

Supplementary Figure 10

Supplementary Figure 11

Supplementary Figure 12

Supplementary Figure 13

Supplementary Figure 14

Supplementary Figure 15

Supplementary Figure 16

Supplementary Figure 17

Supplementary Figure 18

Supplementary Figure 19

Supplementary Figure 20

Supplementary Figure 21

Supplementary Figure 22

Supplementary Figure 23

Supplementary Table 1

Supplementary Table 2

Supplementary Table 3

Supplementary Table 4

Supplementary Table 5

Supplementary Table 6

Supplementary Table 7

Supplementary Table 8

Supplementary Table 9

Supplementary Table 10

## Data Availability

Virus genomes generated here are available from GenBank (accession numbers MW588037 - MW588200), and their respective accession numbers can be found in the Table S5. Metagenomics datasets have been deposited on the SRA database of NCBI with the accession numbers SRR13307418 - SRR13307623 under the BioProject PRJNA685791. Scripts are available at https://github.com/SarahFrancois/SwanMetagenomics.
