# Supplementary material for: Impact of host age on viral and bacterial communities in a waterbird population
Source: ISME J. 2022 Nov 1;17(2):215–26. doi: 10.1038/s41396-022-01334-4 (PMC9860062; doi:10.1038/s41396-022-01334-4)
Supplement: Supplementary file 1 — Supplementary Material [file 41396_2022_1334_MOESM1_ESM.docx]

# Supplementary Information

**
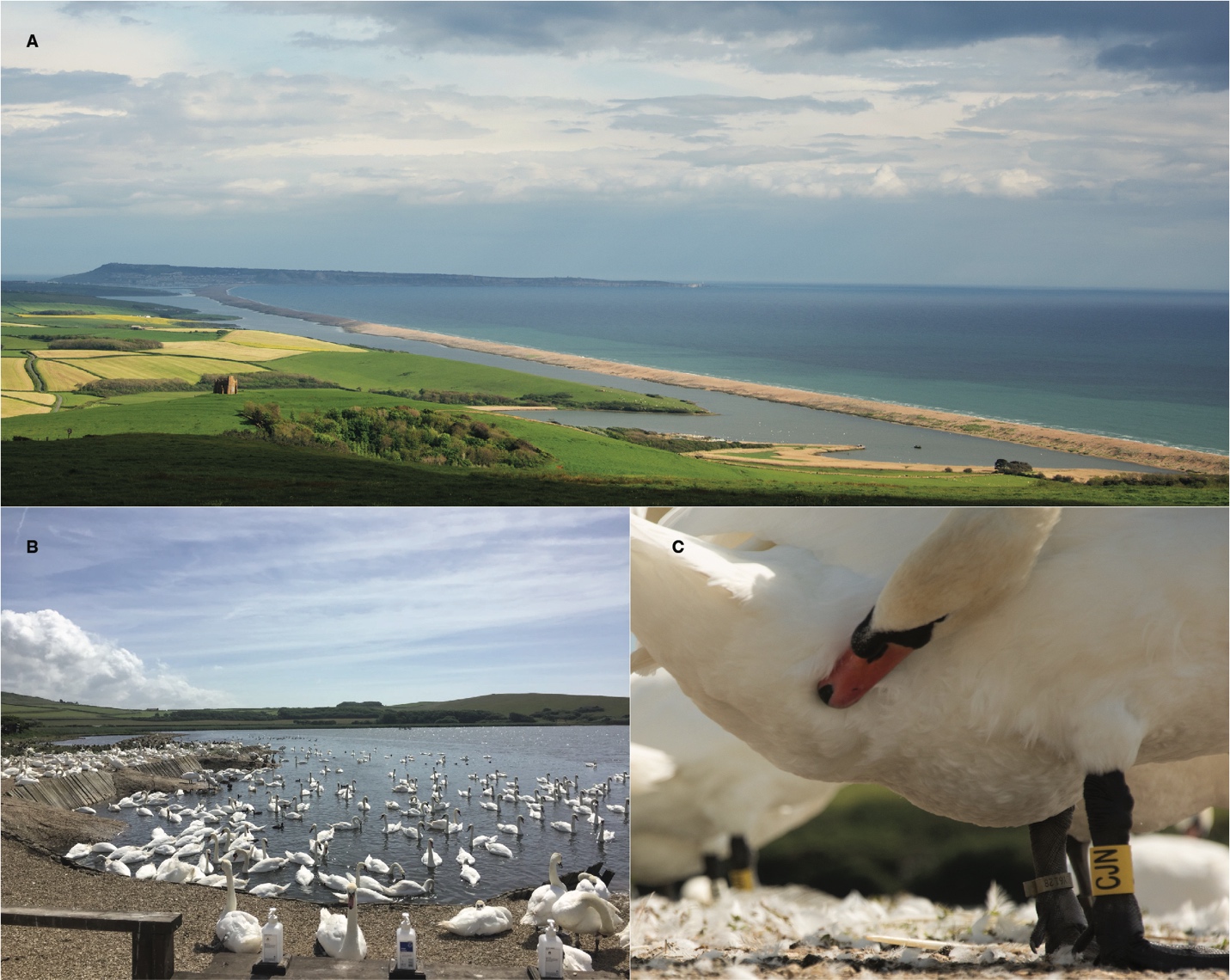
**

**Figure S1:** Photographs of the study population showing **A:** the location of the lagoon next to the sea, viewed towards the southeast. Samples were collected from swans on the shingle beach visible at the lower right edge of the photograph, **B:** the mute swan population at the location of sampling, and **C:** adult swan with darvic (yellow plastic) and British Trust for Ornithology (BTO) (metal) identifier rings. Credit: Mr C. Wheeler (www.wheeler-photography.co.uk).


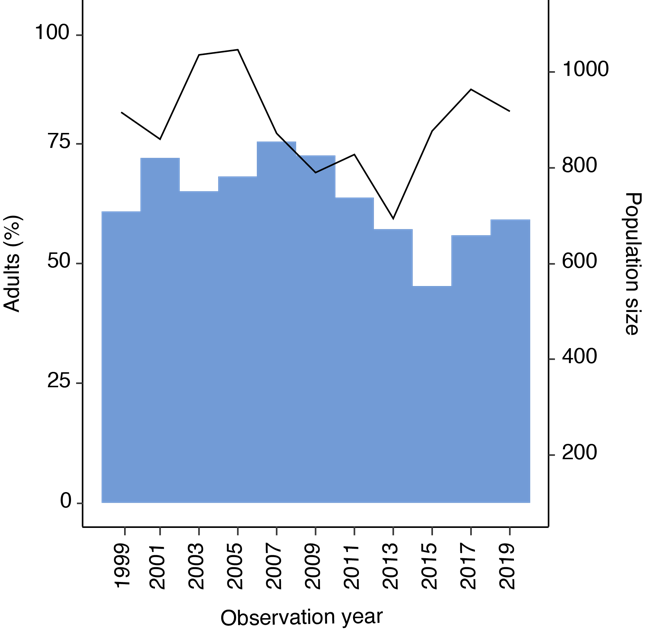


**Figure S2**: Number of individuals in the mute swan population (lines) and frequency of each age groups (bars) from 1999 to 2019.


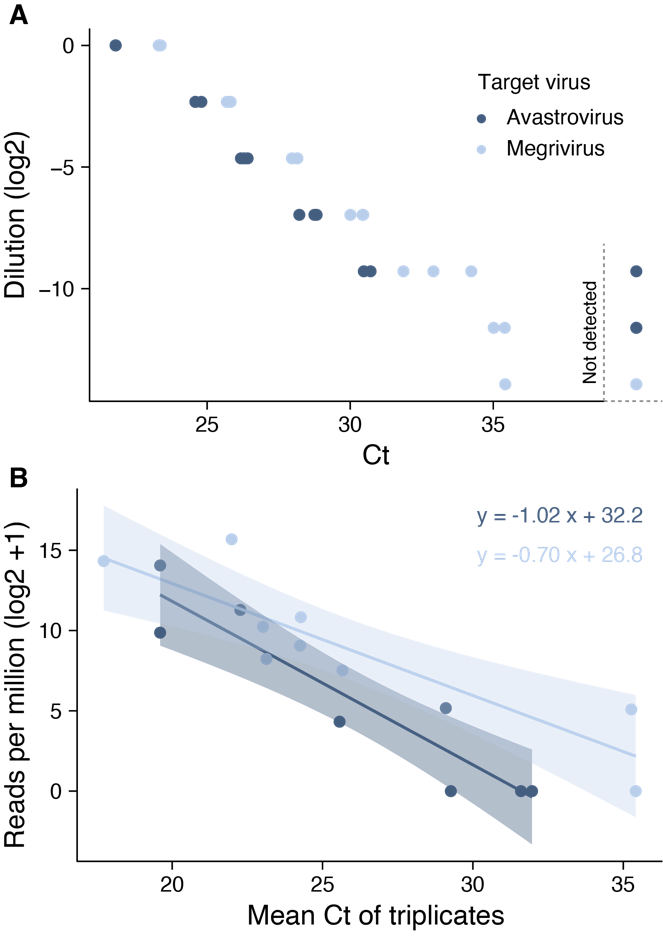


**Figure S3**: (A) Limit of detection of SYBR assays, using 1/5 dilution series completed in triplicate, and (B) qPCR Cts compared to read counts per million for the megrivirus (blue) and avastrovirus assays (red), shown for all samples with detectable Cts. Equations derived from linear modelling are given in the top right corner. 95% confidence intervals for the linear model fit are shown by coloured ribbons.

**
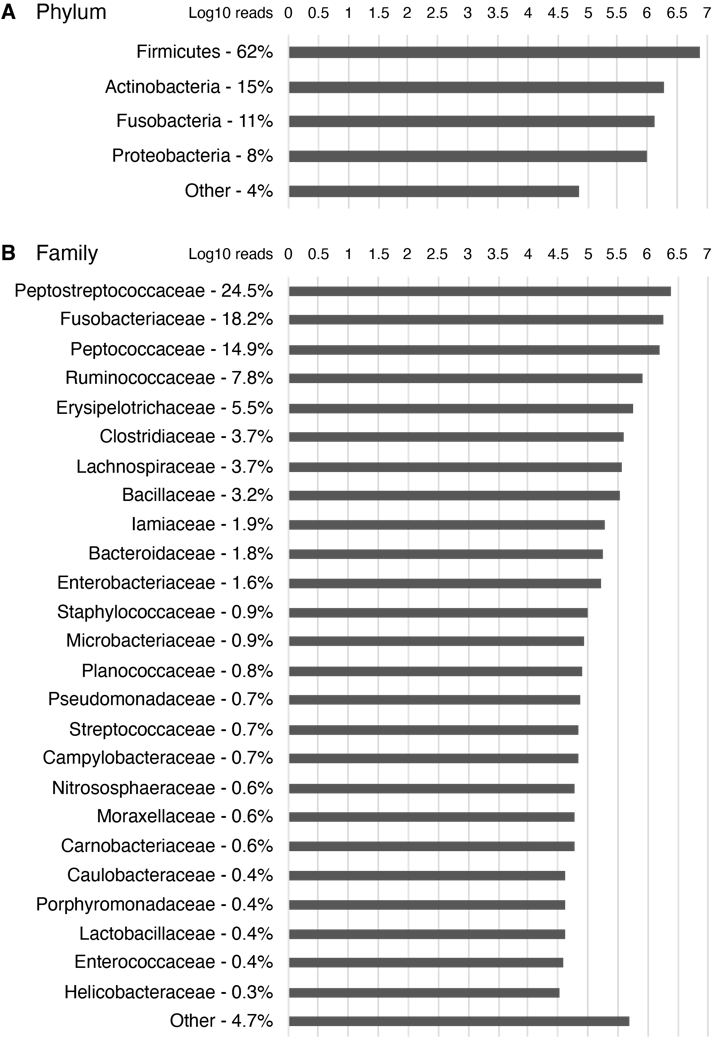
**

**Figure S4**: Observed (A) phyla, and (B) families of *C. olor* faeces core prokaryotic microbiota. Only those taxa present in all samples are considered here as belonging to the core microbiota and other taxa are not displayed. The horizontal (x) axis represents the log10 mean number of 16S read counts attributed to each taxon. For each taxon, the written percentage at the y-axis represents the proportion of 16S counts attributed to each taxon.


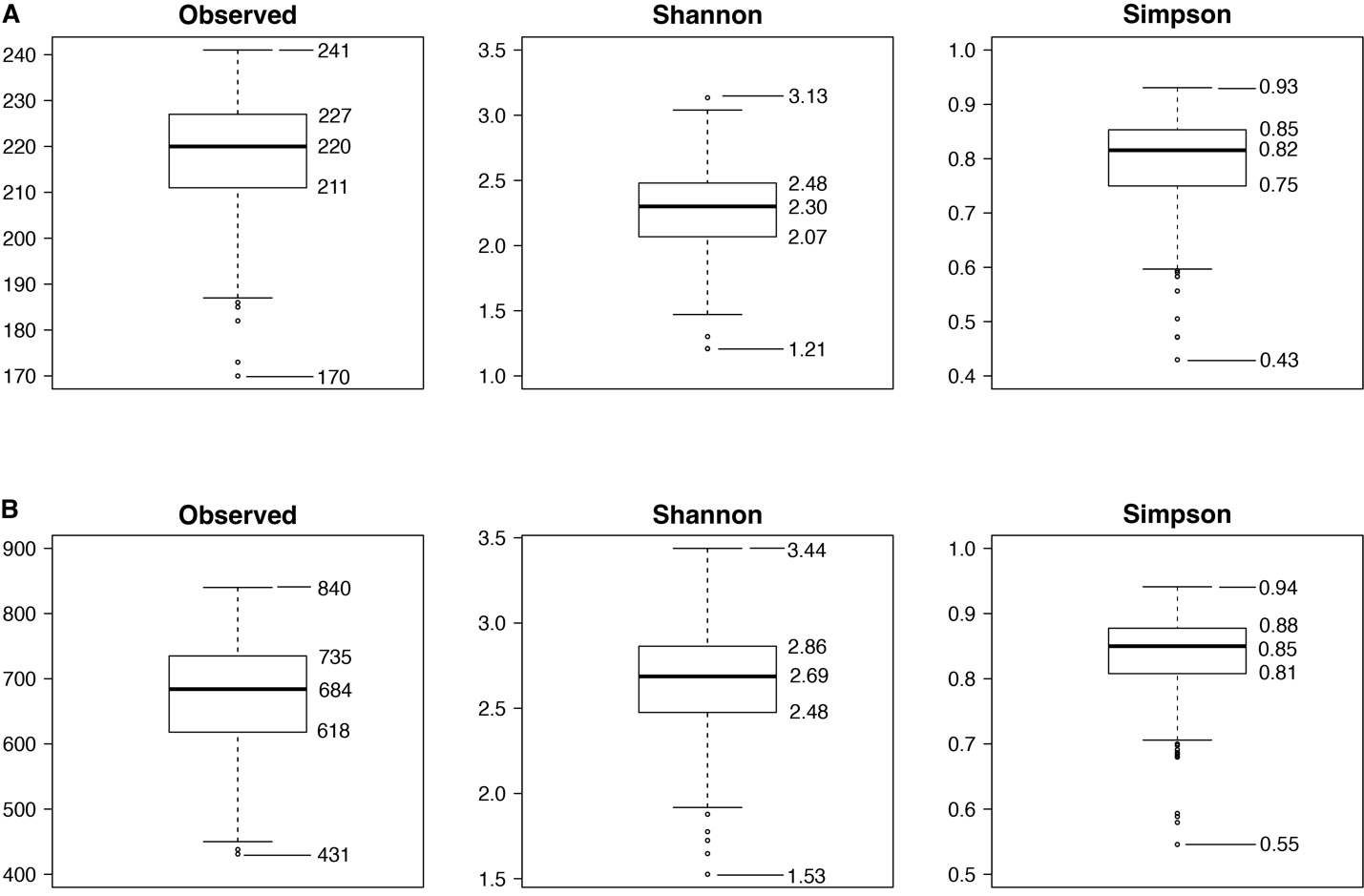


**Figure S5**: Diversity analysis of *C. olor* faecal prokaryotic communities at the (A) family and (B) genus scales. Boxplots indicate the observed numbers, Shannon diversity and Simpson diversity (left to right).


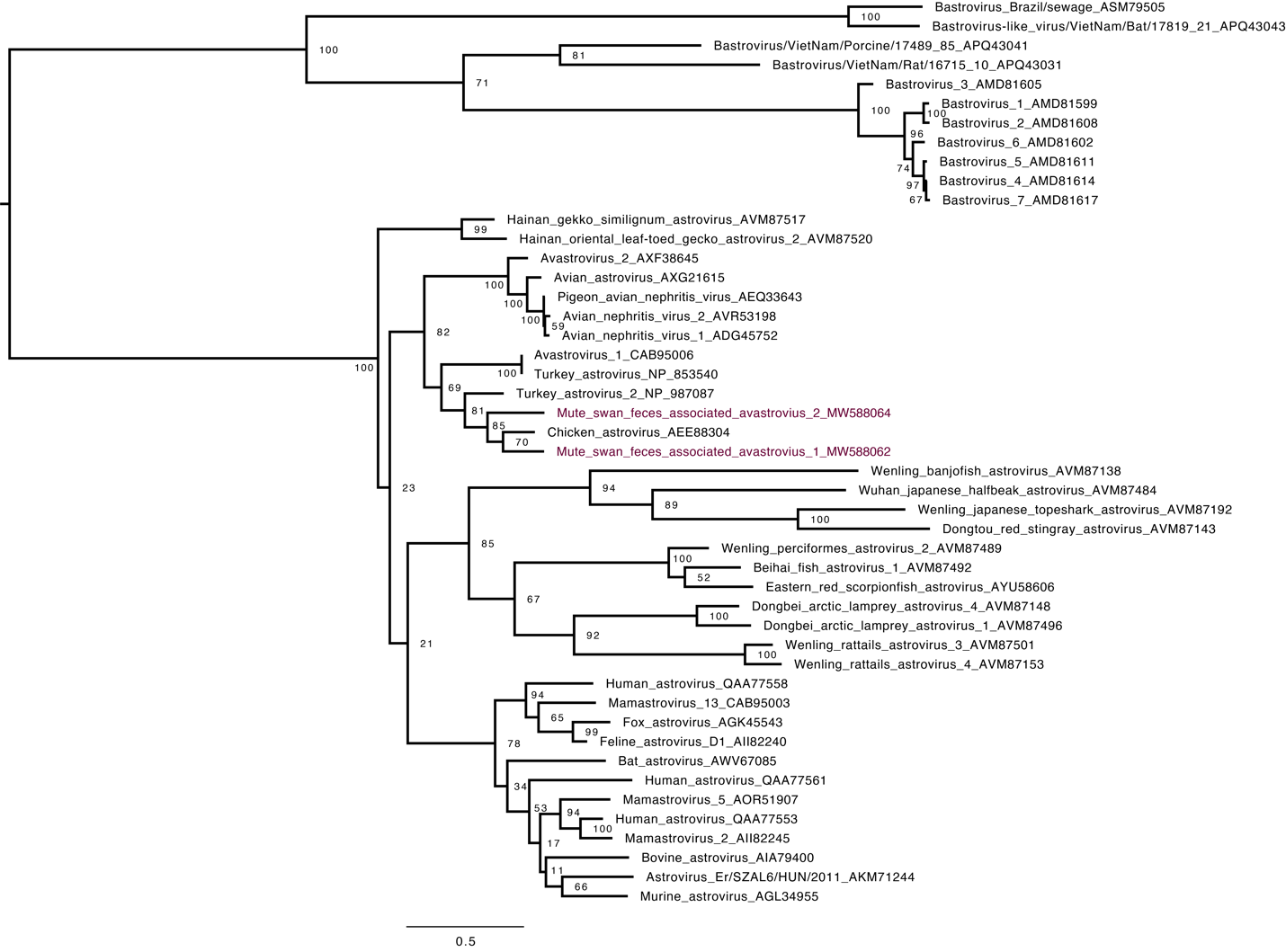


**Figure S6**: ﻿Maximum likelihood phylogenetic tree based on the polymerase protein of 47 taxa of *Astroviridae*. Viruses reported in this study are marked in red. The alignment of 356 amino acids in length was produced using MUSCLE 3.8.425 (16 iterations). The alignment was manually trimmed to remove sites with gaps. The tree was mid-point rooted. Bootstrap values (1000 replicates) are indicated at each node. Scale bar corresponds to amino acid substitutions per site.


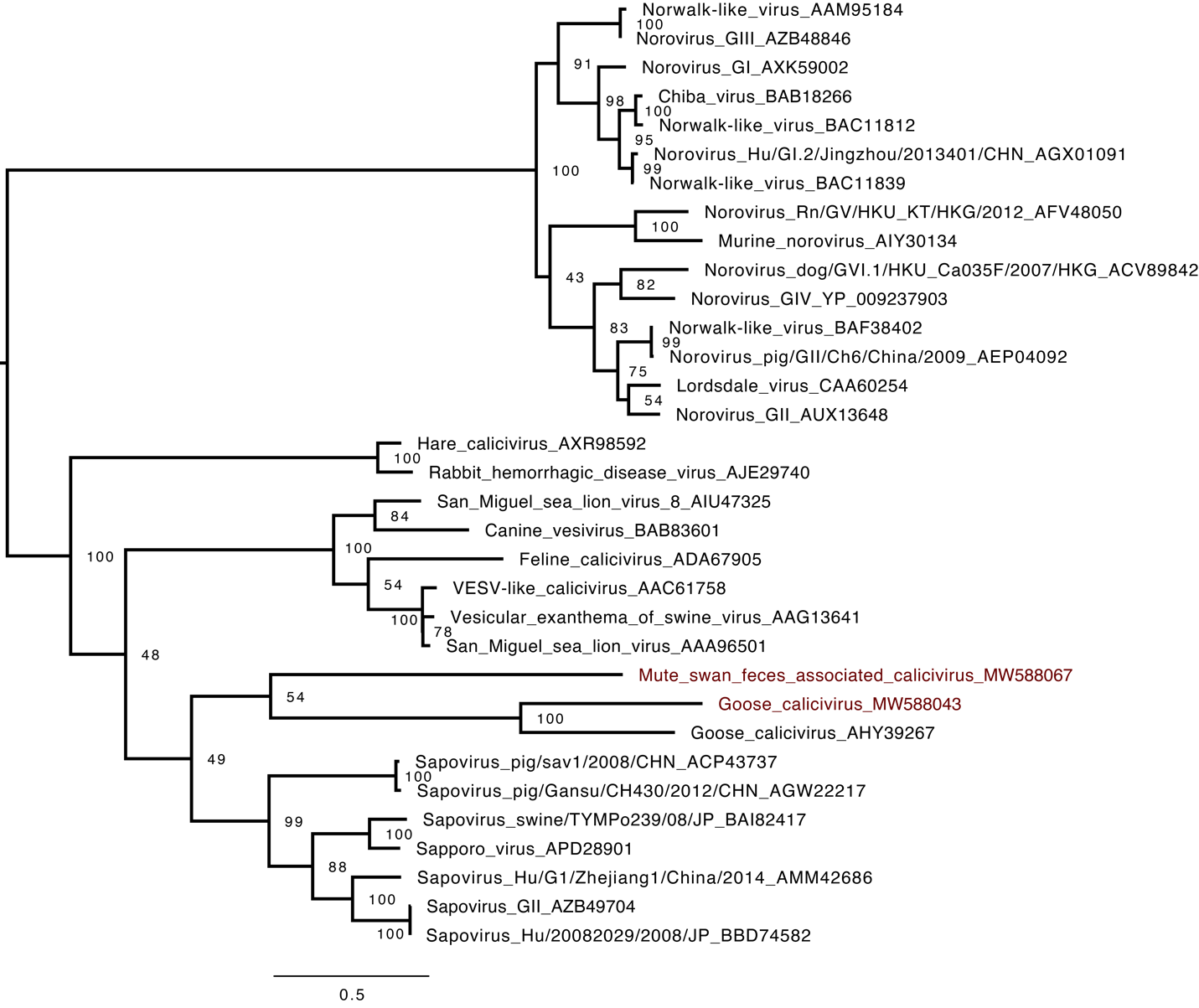


**Figure S7**: ﻿Maximum likelihood phylogenetic tree based on the polymerase protein of 33 taxa of *Caliciviridae*. Viruses reported in this study are marked in red. The alignment of 544 amino acids in length was produced using MUSCLE 3.8.425 (16 iterations). The alignment was manually trimmed to remove sites with gaps. The tree was mid-point rooted. Bootstrap values (1000 replicates) are indicated at each node. Scale bar corresponds to amino acid substitutions per site.


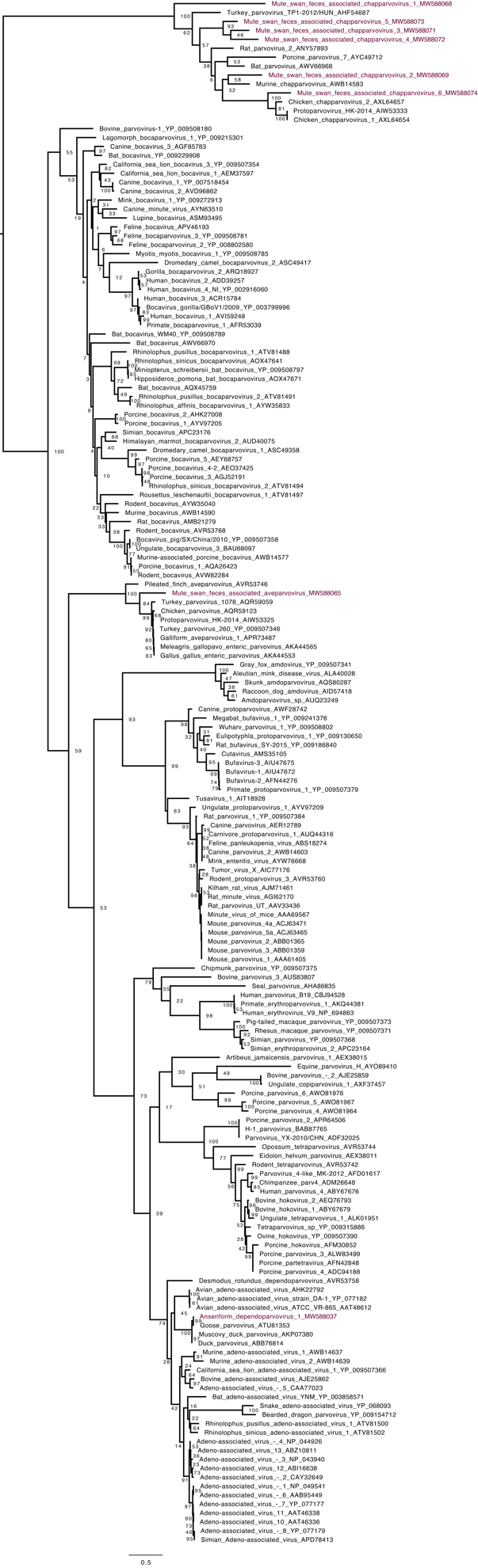


**Figure S8**: ﻿Maximum likelihood phylogenetic tree based on the polymerase protein of 173 taxa of *Parvovirinae*. Viruses reported in this study are marked in red. The alignment of 222 amino acids in length was produced using MUSCLE 3.8.425 (16 iterations). The alignment was manually trimmed to remove sites with gaps. The tree was mid-point rooted. Bootstrap values (1000 replicates) are indicated at each node. Scale bar corresponds to amino acid substitutions per site.


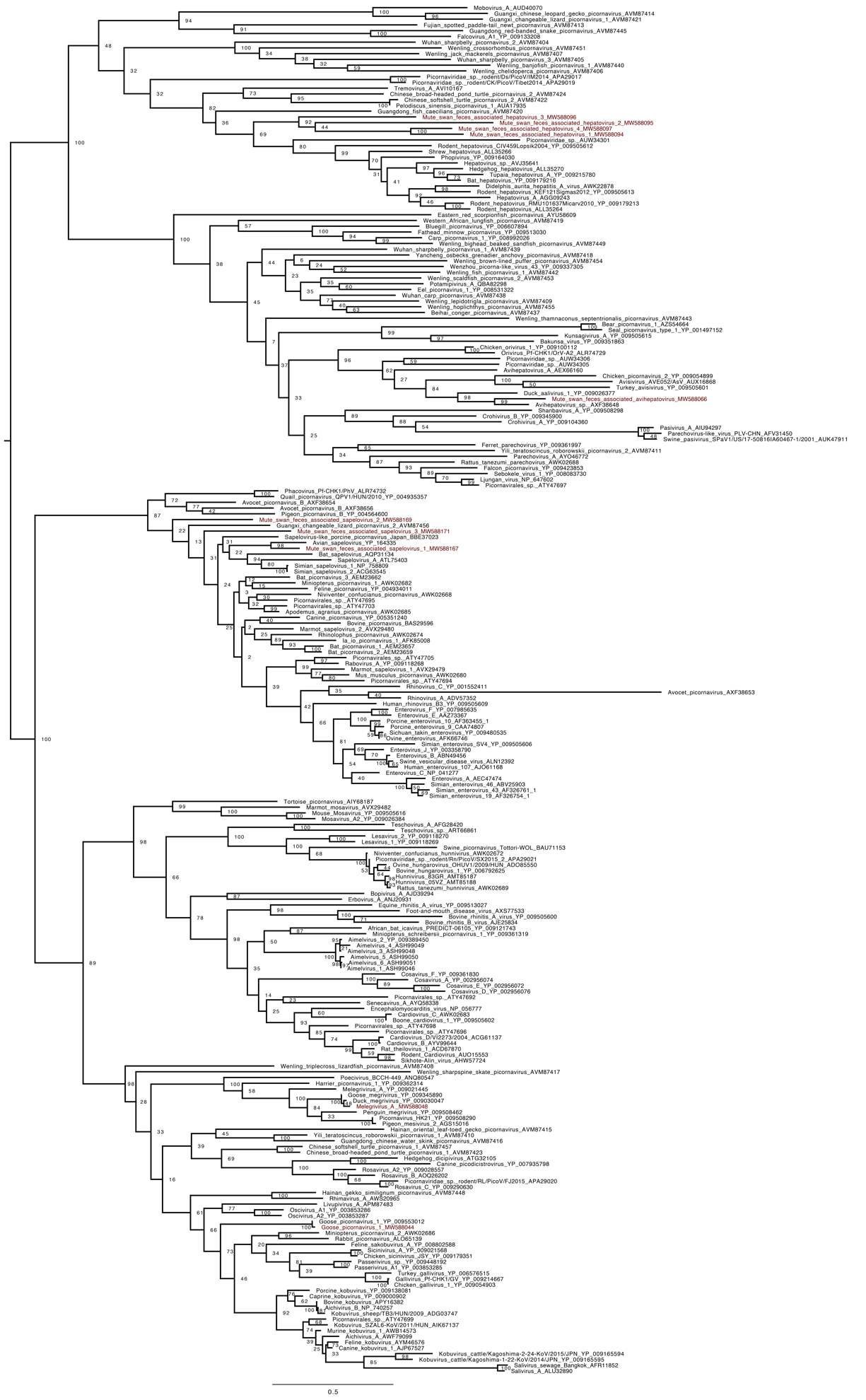


**Figure S9**: ﻿Maximum likelihood phylogenetic tree based on the 3D domain of the polymerase protein of 238 taxa of *Picornaviridae*. Viruses reported in this study are marked in red. The alignment of 418 amino acids in length was produced using MAFFT v7.388. The alignment was manually trimmed to remove sites with gaps. The tree was mid-point rooted. Bootstrap values (1000 replicates) are indicated at each node. Scale bar corresponds to amino acid substitutions per site.


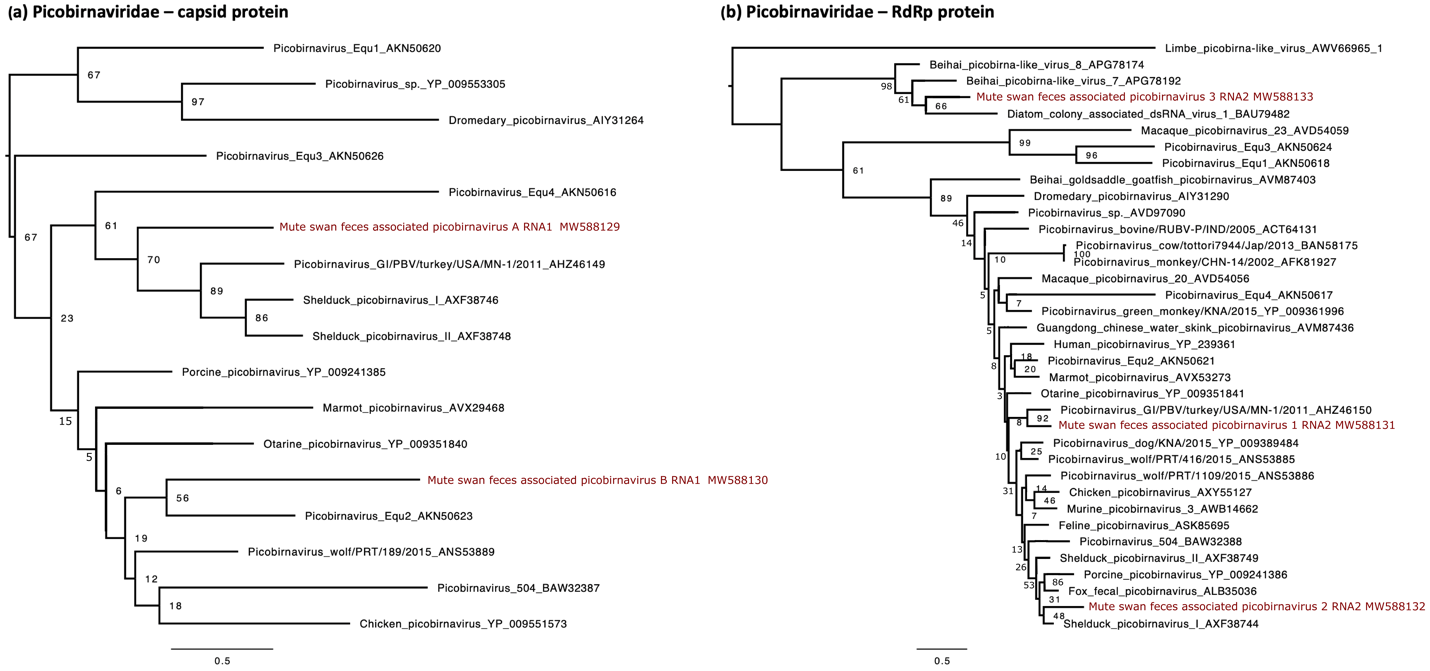


**Figure S10**: ﻿Maximum likelihood phylogenetic tree based on (a) the capsid protein of 17 taxa of *Picobirnaviridae*, with an alignment of 172 amino acids in length; (b) the polymerase protein of 36 taxa of *Picobirnaviridae*, with an alignment of 333 amino acids in length. Viruses reported in this study are marked in red. The alignments were produced using MUSCLE 3.8.425 (16 iterations). The alignment was manually trimmed to remove sites with gaps. The tree was mid-point rooted. Bootstrap values (1000 replicates) are indicated at each node. Scale bar corresponds to amino acid substitutions per site.


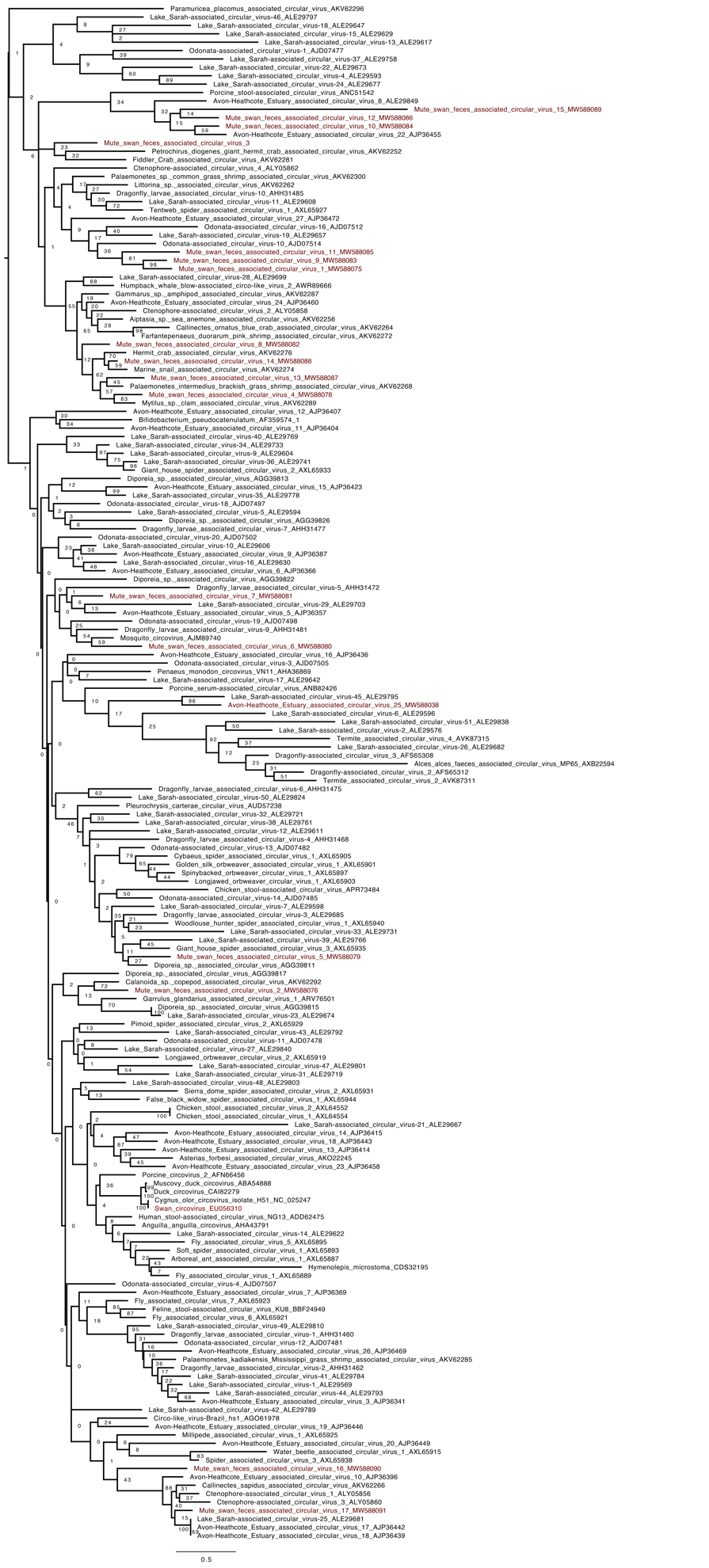


**Figure S11**: ﻿Maximum likelihood phylogenetic tree based on the polymerase protein of 183 *Circoviridae* taxa. Viruses reported in this study are marked in red. The alignment of 285 amino acids in length was produced using MUSCLE 3.8.425 (16 iterations). The alignment was manually trimmed to remove sites with gaps. The tree was mid-point rooted. Bootstrap values (1000 replicates) are indicated at each node. Scale bar corresponds to amino acid substitutions per site.


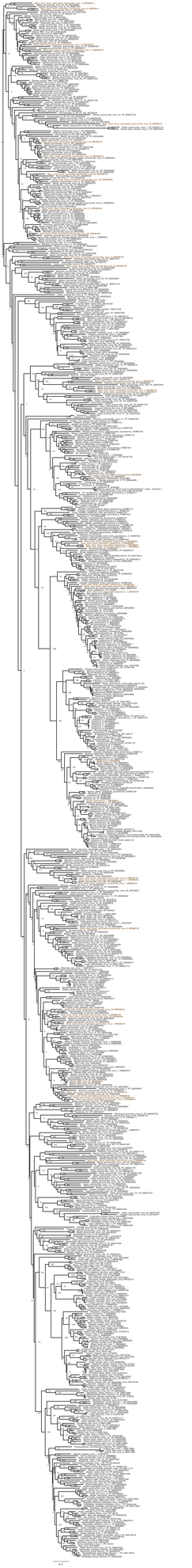


**Figure S12**: ﻿Maximum likelihood phylogenetic tree based on the polymerase protein of 952 *Picornavirales* taxa. Viruses reported in this study are marked in red. The alignment of 1235 amino acids in length was produced using MAFFT v7.388. The alignment was manually trimmed to remove sites with gaps. The tree was mid-point rooted. Bootstrap values (1000 replicates) are indicated at each node. Scale bar corresponds to amino acid substitutions per site.


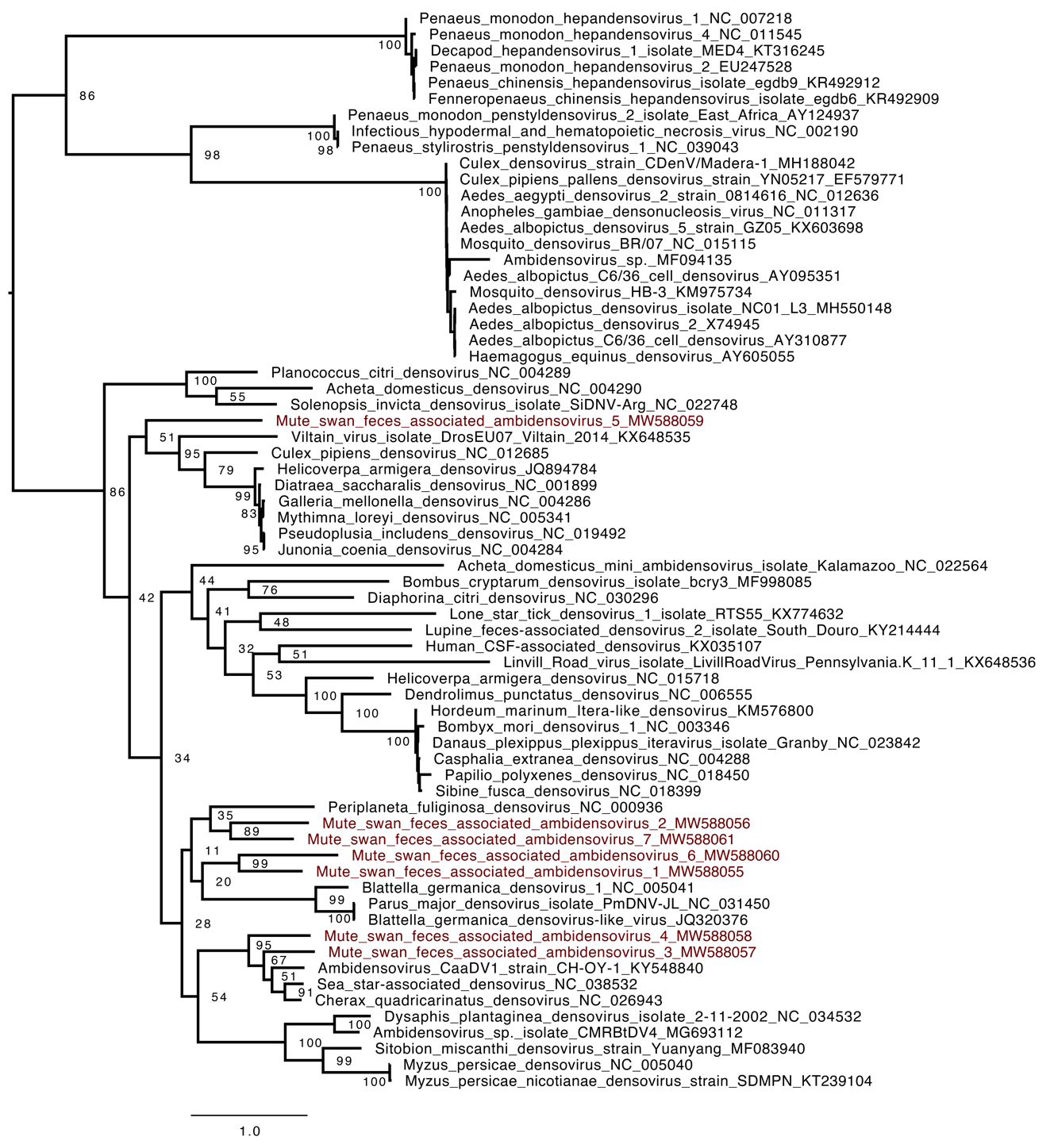


**Figure S13**: ﻿Maximum likelihood phylogenetic tree based on the polymerase protein of 67 *Densovirinae* taxa. Viruses reported in this study are marked in red. The alignment of 509 amino acids in length was produced using MUSCLE 3.8.425 (16 iterations). The alignment was manually trimmed to remove sites with gaps. The tree was mid-point rooted. Bootstrap values (1000 replicates) are indicated at each node. Scale bar corresponds to amino acid substitutions per site.


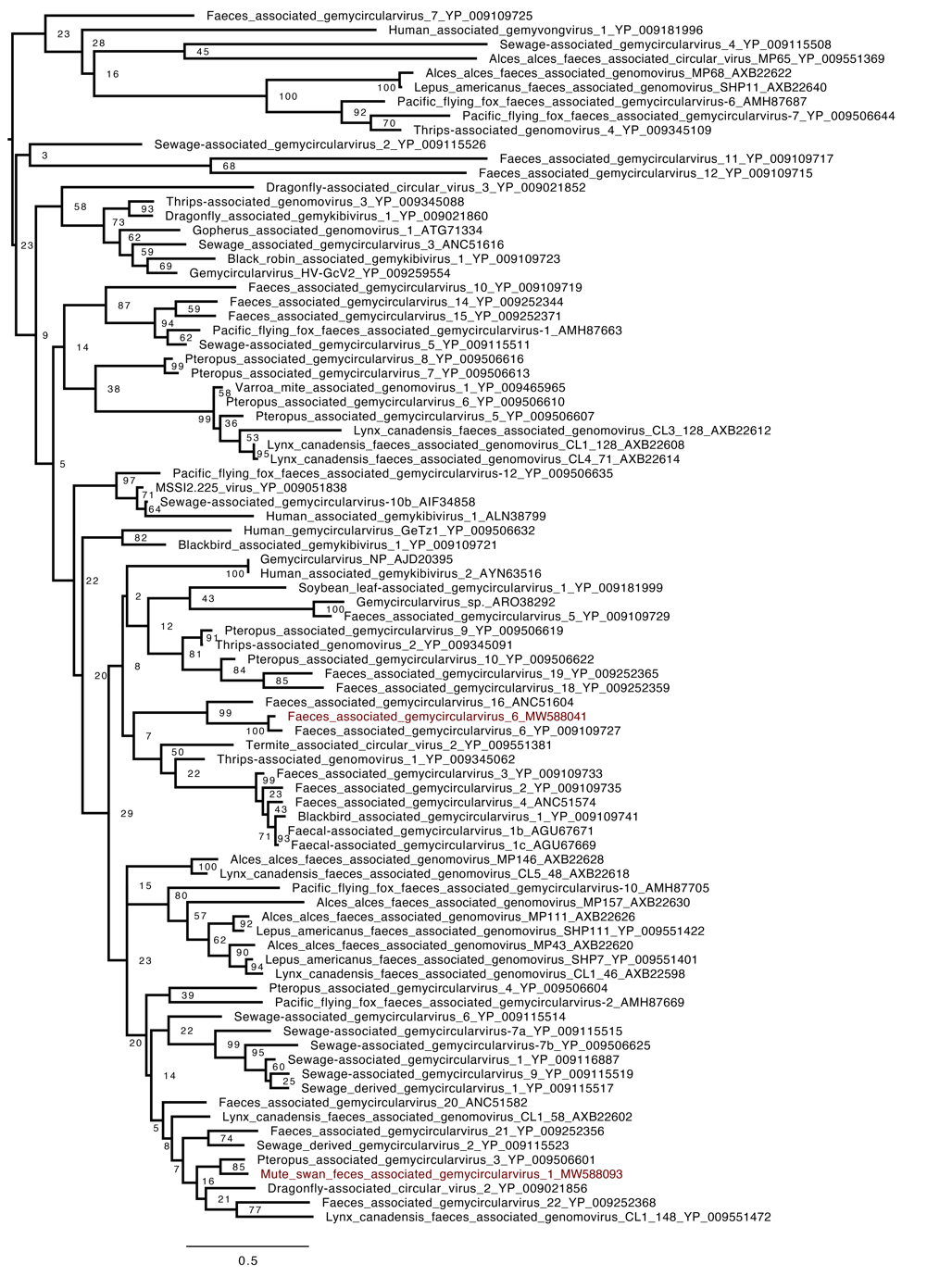


**Figure S14**: ﻿Maximum likelihood phylogenetic tree based on the polymerase protein of 85 *Genomoviridae* taxa. Viruses reported in this study are marked in red. The alignment of 211 amino acids in length was produced using MUSCLE 3.8.425 (16 iterations). The alignment was manually trimmed to remove sites with gaps. The tree was mid-point rooted. Bootstrap values (1000 replicates) are indicated at each node. Scale bar corresponds to amino acid substitutions per site.


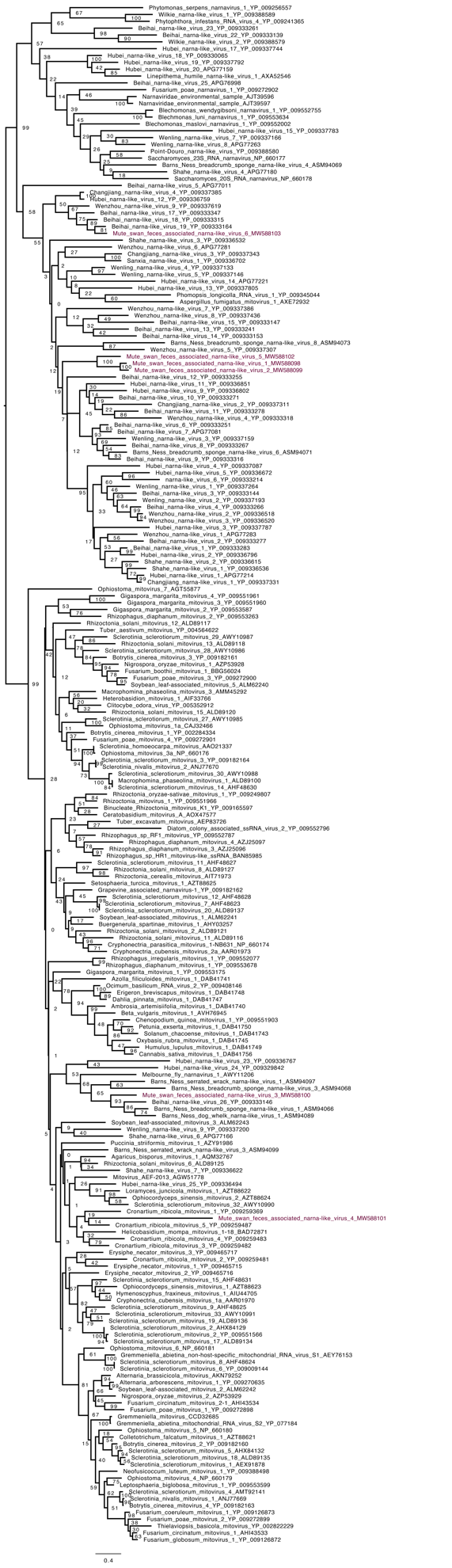


**Figure S15**: ﻿Maximum likelihood phylogenetic tree based on the polymerase protein of 225 *Narnaviridae* taxa. Viruses reported in this study are marked in red. The alignment of 565 amino acids in length was produced using MUSCLE 3.8.425 (16 iterations). The alignment was manually trimmed to remove sites with gaps. The tree was mid-point rooted. Bootstrap values (1000 replicates) are indicated at each node. Scale bar corresponds to amino acid substitutions per site.

**
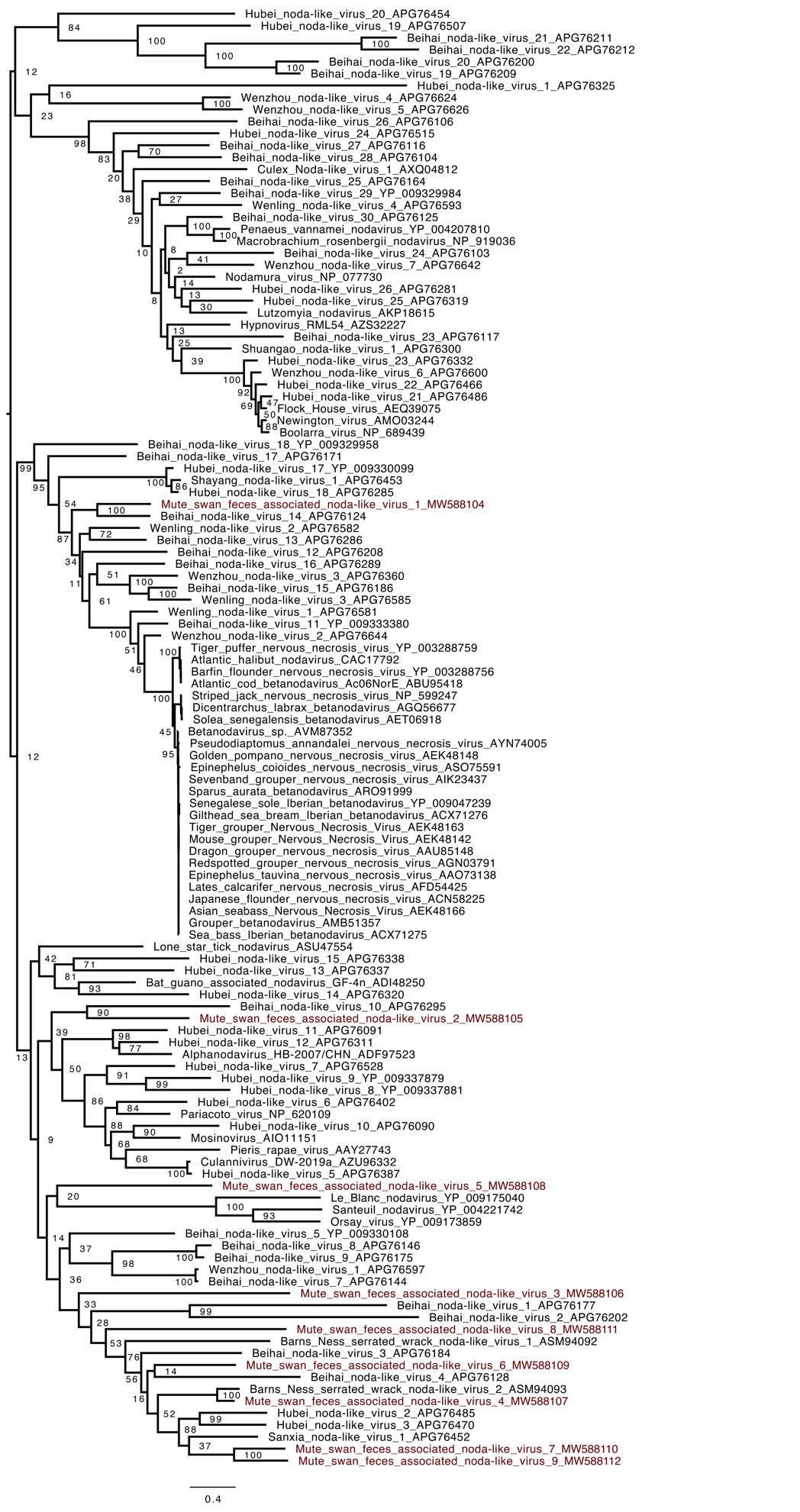
**

**Figure S16**: ﻿Maximum likelihood phylogenetic tree based on the polymerase protein of 122 *Nodaviridae* taxa. Viruses reported in this study are marked in red. The alignment of 1284 amino acids in length was produced using MUSCLE 3.8.425 (16 iterations). The alignment was manually trimmed to remove sites with gaps. The tree was mid-point rooted. Bootstrap values (1000 replicates) are indicated at each node. Scale bar corresponds to amino acid substitutions per site.


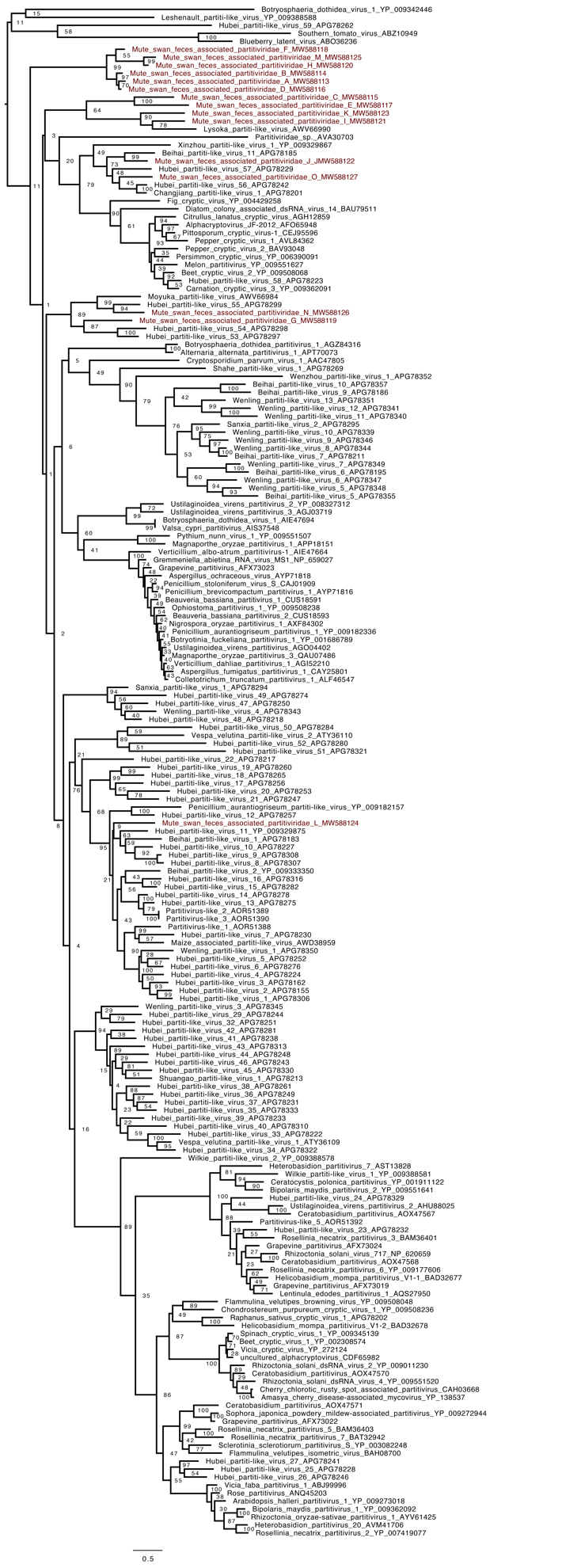


**Figure S17**: ﻿Maximum likelihood phylogenetic tree based on the polymerase protein of 192 *Partitiviridae* taxa. Viruses reported in this study are marked in red. The alignment of 692 amino acids in length was produced using MUSCLE 3.8.425 (16 iterations). The alignment was manually trimmed to remove sites with gaps. The tree was mid-point rooted. Bootstrap values (1000 replicates) are indicated at each node. Scale bar corresponds to amino acid substitutions per site.


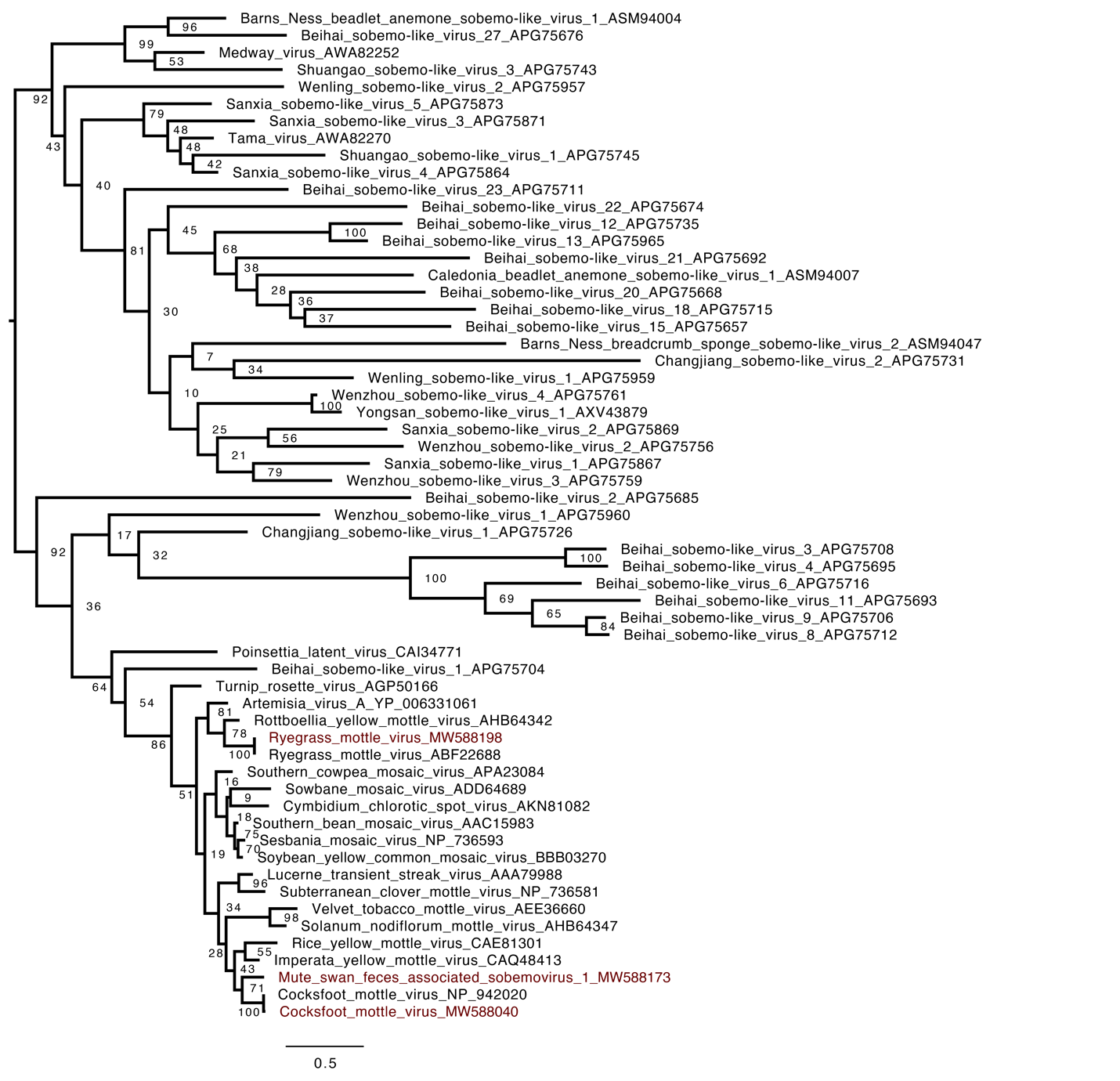


**Figure S18**: ﻿Maximum likelihood phylogenetic tree based on the polymerase protein of 59 *Sobemovirus* taxa. Viruses reported in this study are marked in red. The alignment of 251 amino acids in length was produced using MUSCLE 3.8.425 (16 iterations). The alignment was manually trimmed to remove sites with gaps. The tree was mid-point rooted. Bootstrap values (1000 replicates) are indicated at each node. Scale bar corresponds to amino acid substitutions per site.


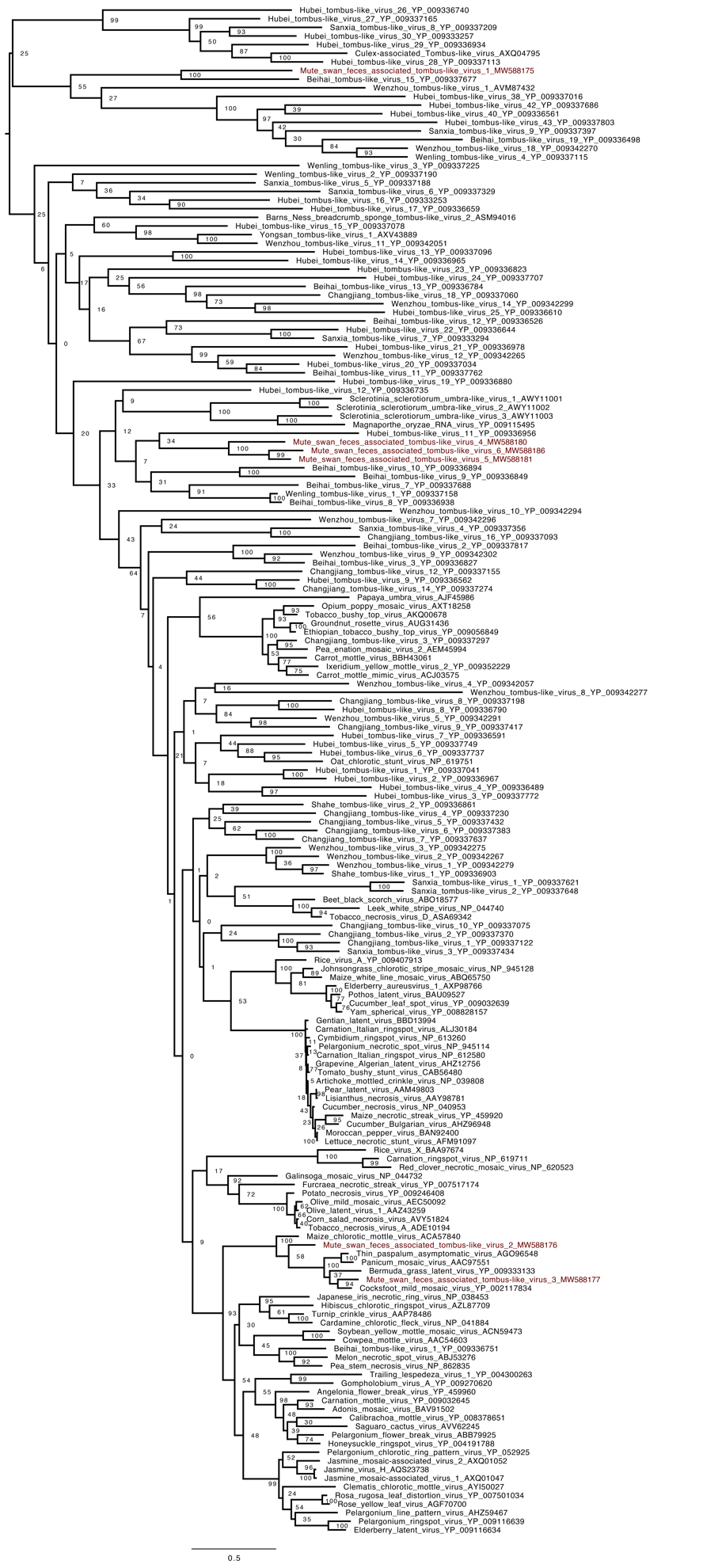


**Figure S19**: ﻿Maximum likelihood phylogenetic tree based on the polymerase protein of 177 *Tombusviridae* taxa. Viruses reported in this study are marked in red. The alignment of 411 amino acids in length was produced using MUSCLE 3.8.425 (16 iterations). The alignment was manually trimmed to remove sites with gaps. The tree was mid-point rooted. Bootstrap values (1000 replicates) are indicated at each node. Scale bar corresponds to amino acid substitutions per site.


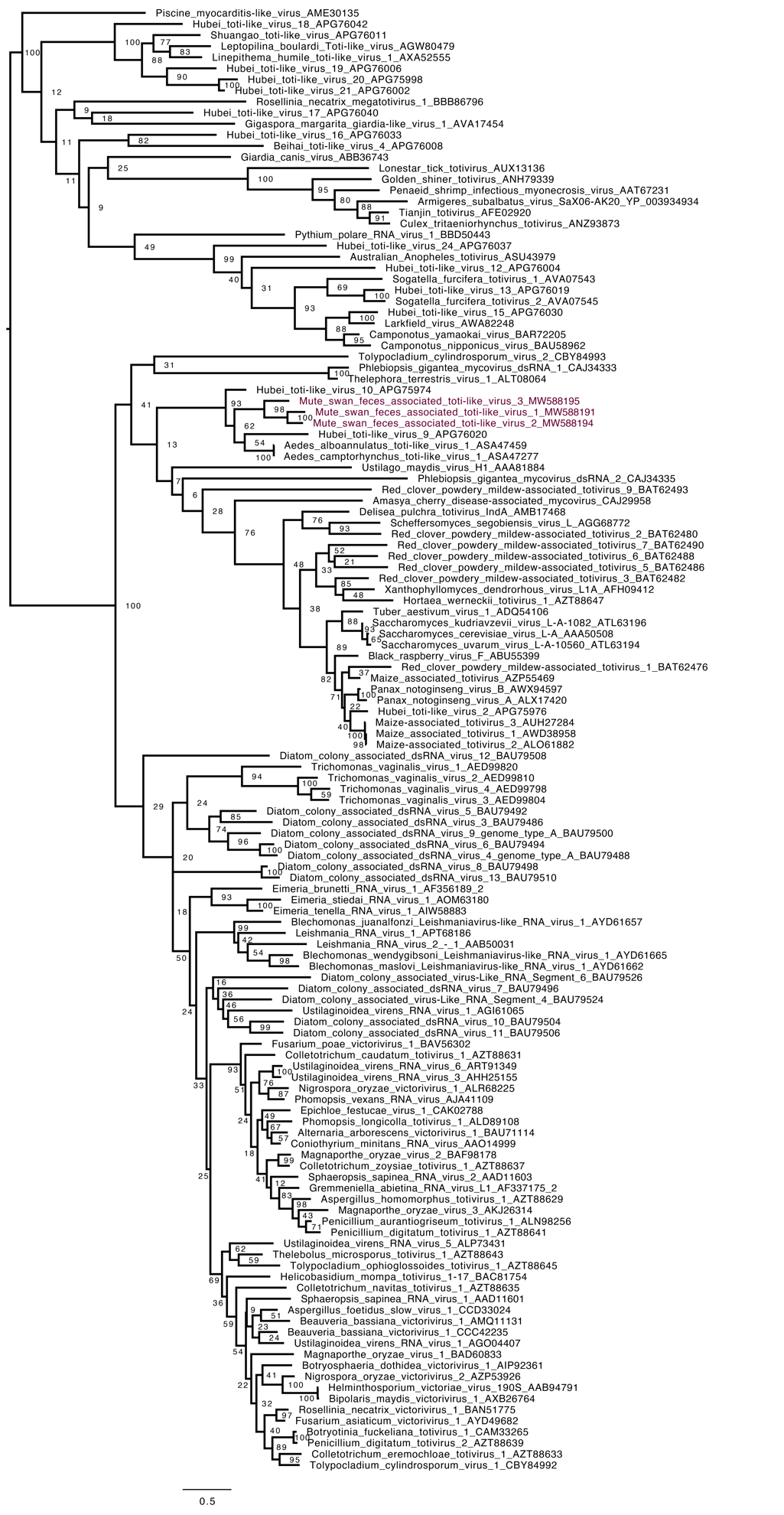


**Figure S20**: ﻿Maximum likelihood phylogenetic tree based on the polymerase protein of 132 *Totiviridae* taxa. Viruses reported in this study are marked in red. The alignment of 343 amino acids in length was produced using MUSCLE 3.8.425 (16 iterations). The alignment was manually trimmed to remove sites with gaps. The tree was mid-point rooted. Bootstrap values (1000 replicates) are indicated at each node. Scale bar corresponds to amino acid substitutions per site.


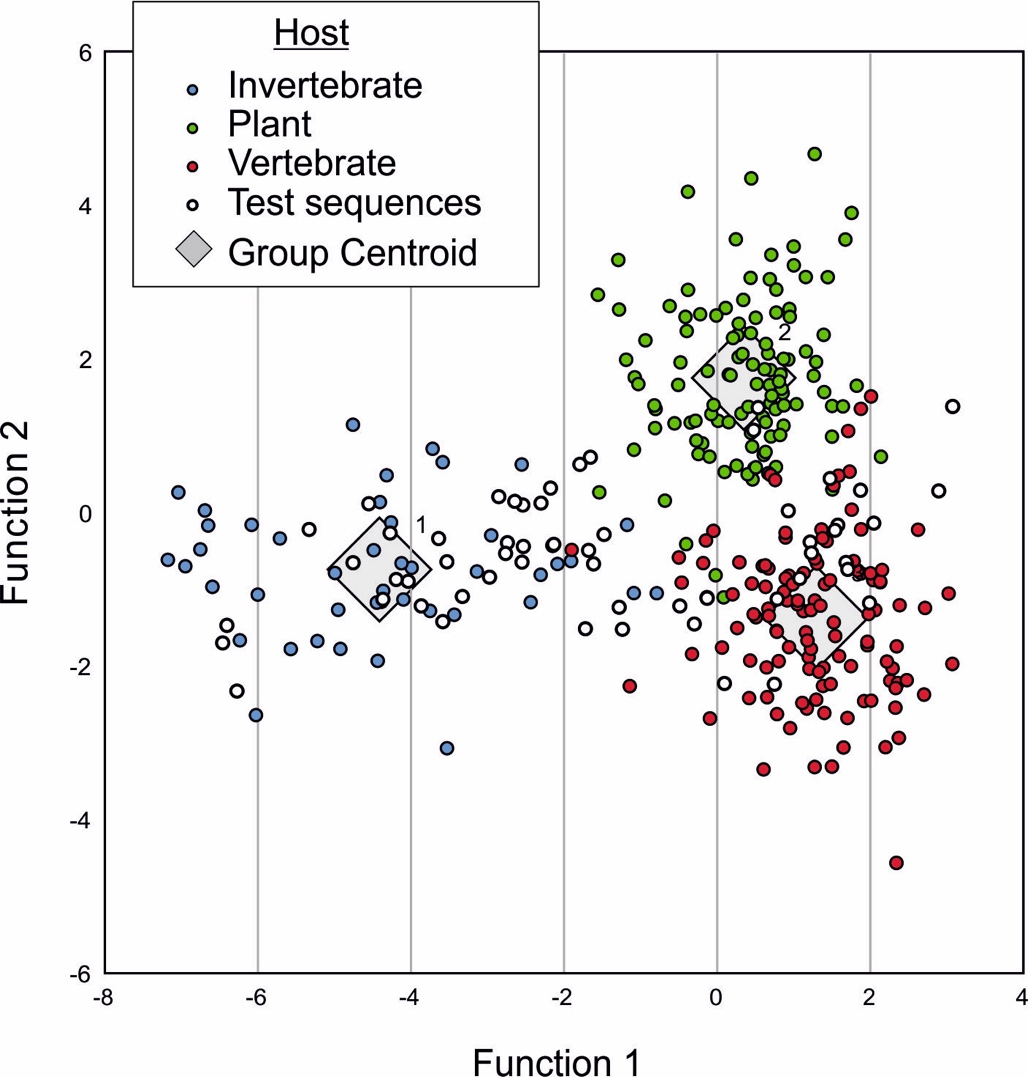


**Figure S21:** Canonical score plot of discriminant analysis used to classify viral sequences by hosts (vertebrate, invertebrate, plant) using mononucleotide and dinucleotide frequencies. The axes represent the two most influential factors. Coloured points represent values for individual sequences in the reference dataset for which hosts are known, and white points represent the virus contigs recovered here. Diamonds indicate the centroid of points from each known host category.


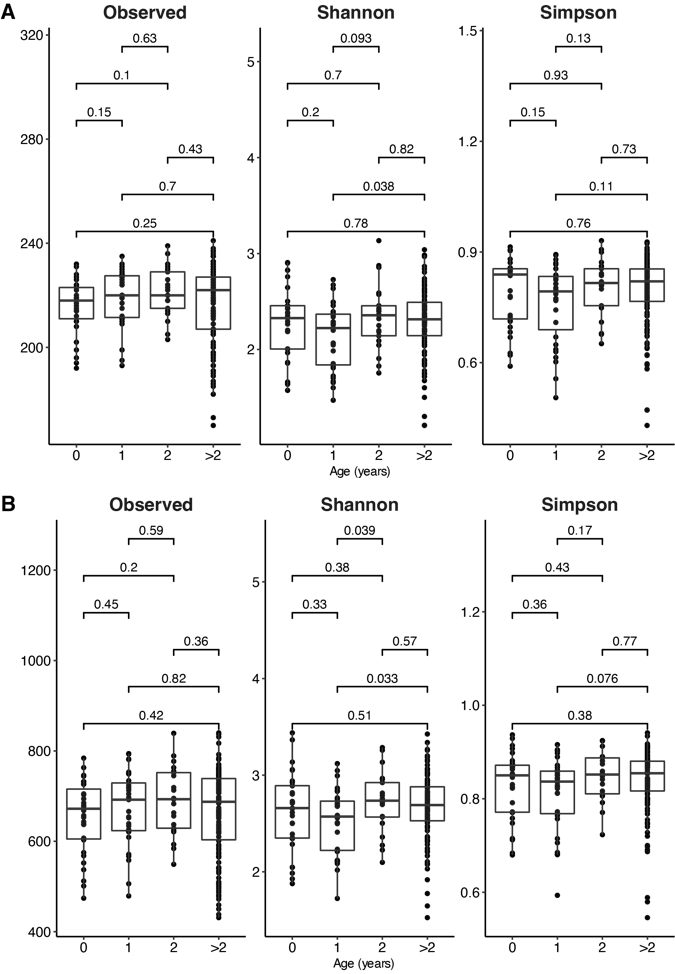


**Figure S22**: Pairwise comparisons of the impact of bird age on *C. olor* faecal prokaryotic communities’ diversity at the family scale (A) and the genus scale (B). Diversity analysis comprises observed richness (i.e. the total number of taxa present per sample), Shannon and Simpson indexes. The numbers indicate *p* values.


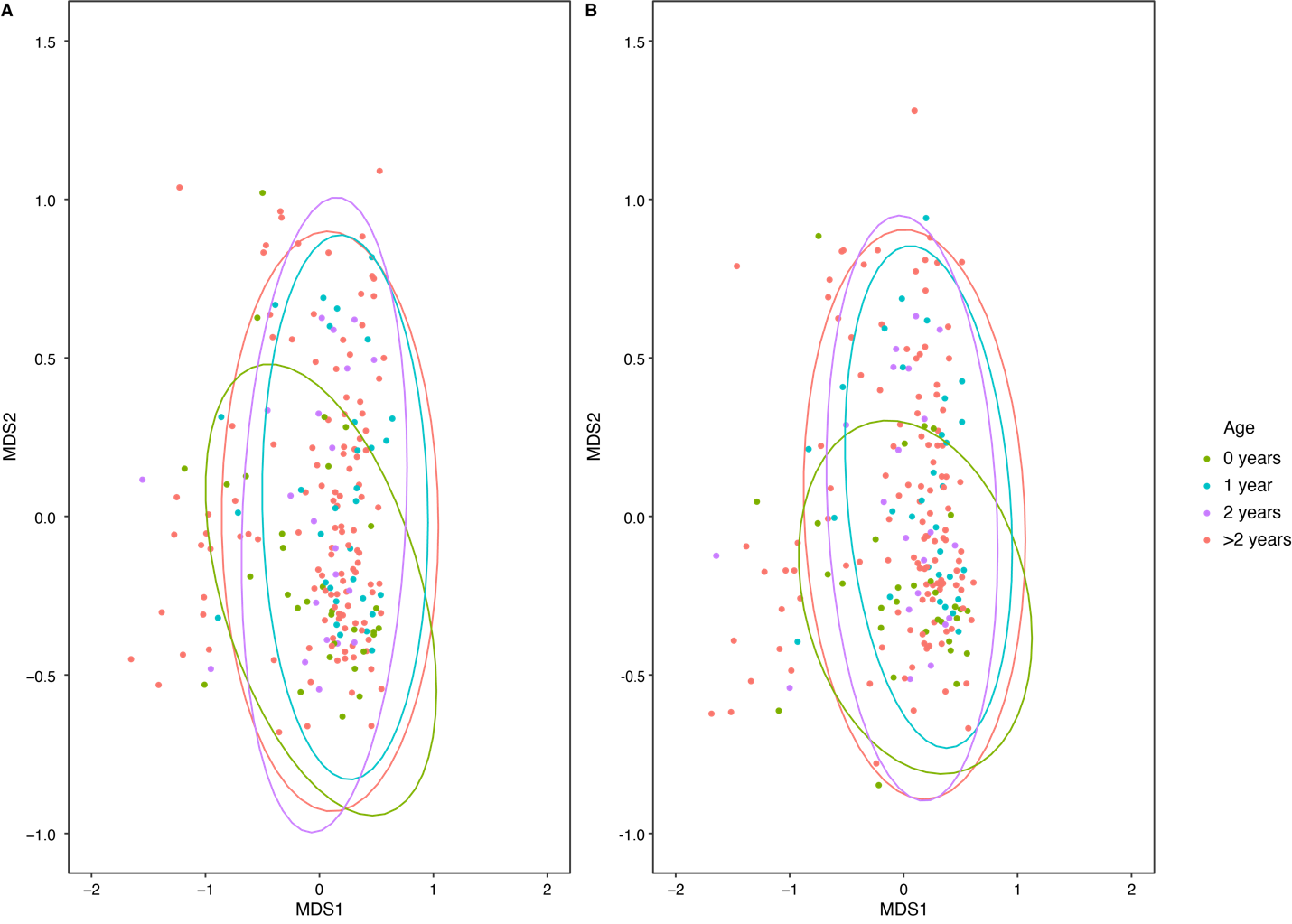


**Figure S23**: Non-metric multi-dimensional scaling plots of prokaryotes communities based on Bray-Curtis dissimilarity (A) at the family level (stress value: 0.18), (B) at the genus level (stress value: 0.17). Data were not transformed. Samples are coloured by bird age.

**Supplementary Tables are submitted as Supporting Files:**

**Table S1:** Discriminant analyses of virus host range by virus genomic nucleotide composition.

**Table S2**: Primers for SYBR green qPCRs.

**Table S3**: Collected samples and their associated metadata.

**Table S4**: Prokaryotic 16S reads counts contingency tables (domain, phylum, class, order, family and genus scales).

**Table S5**: List of viruses reported in this study, associated information, and accession numbers.

**Table S6**: Viral reads counts contingency tables (family and species scales).

**Table S7**: Reported host range and geographical range of the already known virus species detected in this study.

**Table S8**: Reported swan-infecting (*Cygnus* *spp.*) viruses from PubMed search, conducted on 10/03/2020.

**Table S9**: Effects of bird age, sex, and seasonality on the diversity of prokaryotic communities assessed at the family and genus levels with PERMANOVAs based on Bray–Curtis dissimilarity matrices, and permutational tests of dispersions (PERDISPs). Bold values indicate a significant effect of the tested factor after Bonferroni correction, and stars indicate the significance level of this test (**p* < 0.05, ***p* < 0.01, ****p* < 0.001). PERDISPs showed no significant differences in dispersion between groups (*p* values > 0.05).

**Table S10**: Information on differentially prevalent and/or abundant prokaryote families and genera and virus families and species according to bird age.
