# Supplementary figures and images for: Impact of host age on viral and bacterial communities in a waterbird population
Source: ISME J. 2022 Nov 1;17(2):215–26. doi: 10.1038/s41396-022-01334-4 (PMC9860062; doi:10.1038/s41396-022-01334-4)

A

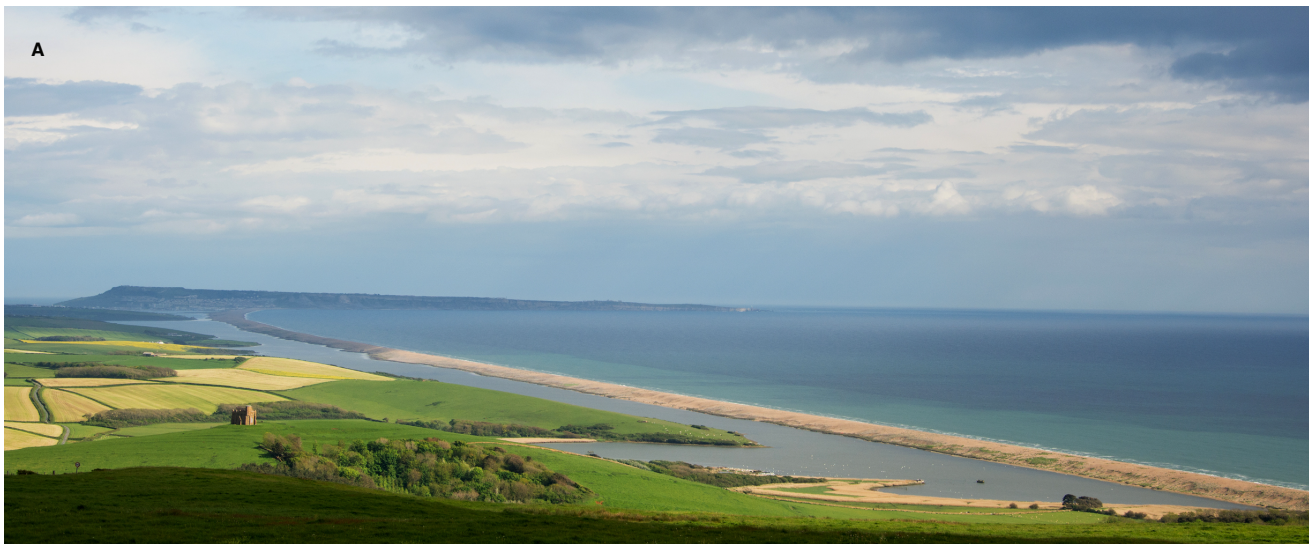

B

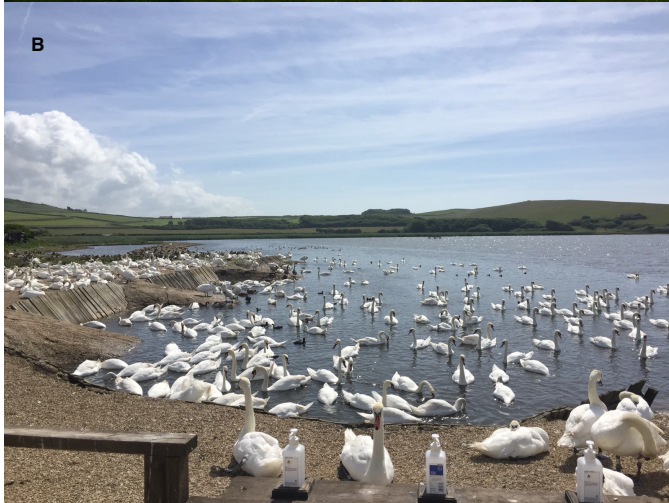

C

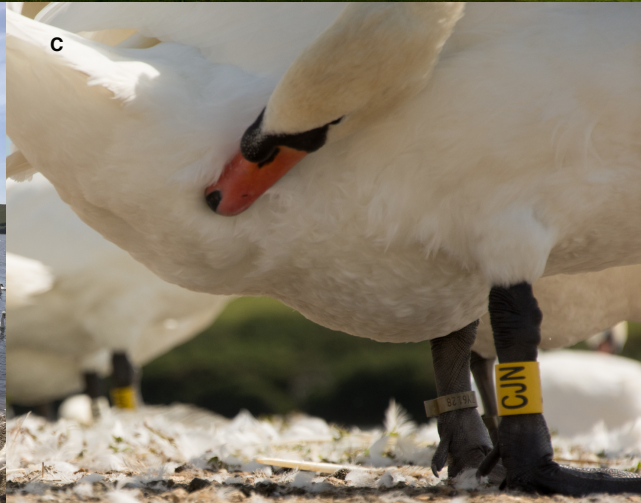

Supplement: Supplementary file 2 — Supplementary Figure 1 [file 41396_2022_1334_MOESM2_ESM.pdf]

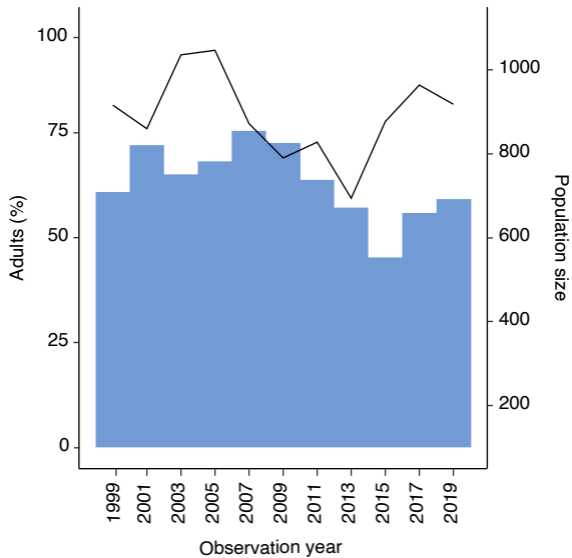

Supplement: Supplementary file 3 — Supplementary Figure 2 [file 41396_2022_1334_MOESM3_ESM.pdf]

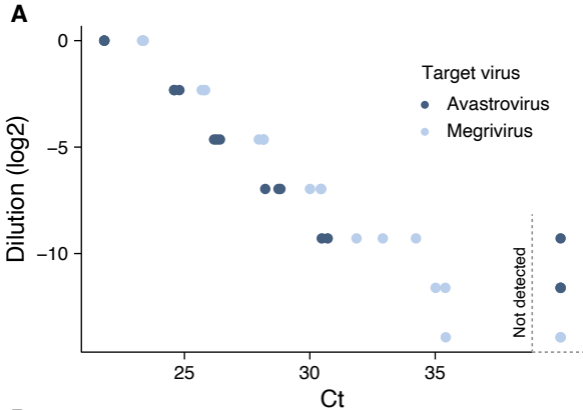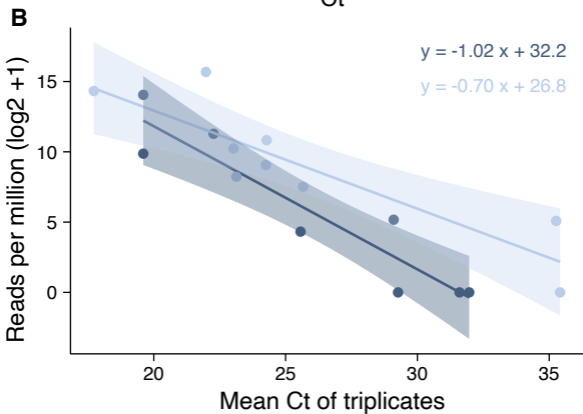

Supplement: Supplementary file 4 — Supplementary Figure 3 [file 41396_2022_1334_MOESM4_ESM.pdf]

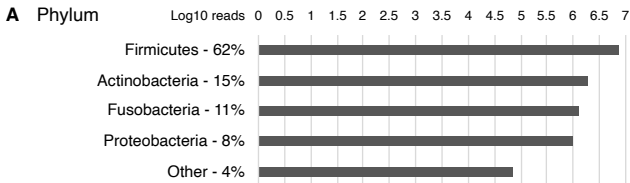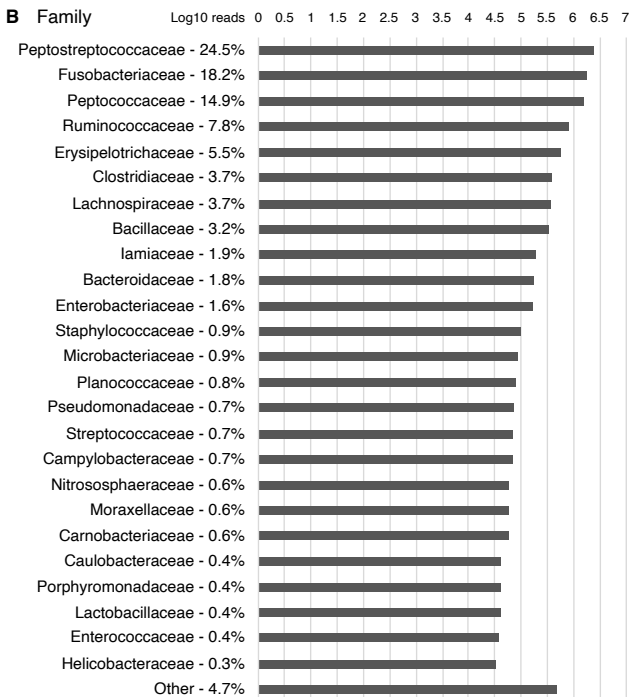

Supplement: Supplementary file 5 — Supplementary Figure 4 [file 41396_2022_1334_MOESM5_ESM.pdf]

**A****Observed**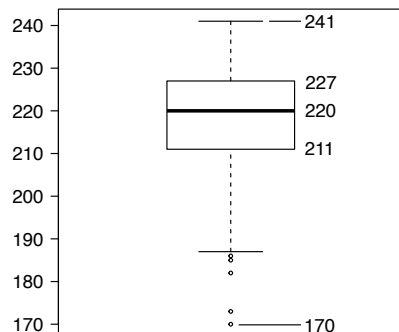**Shannon**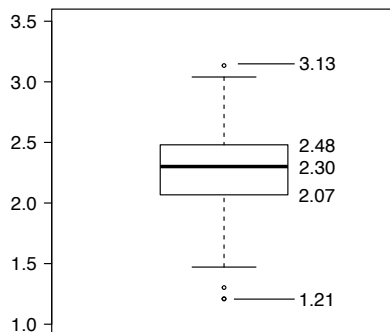**Simpson**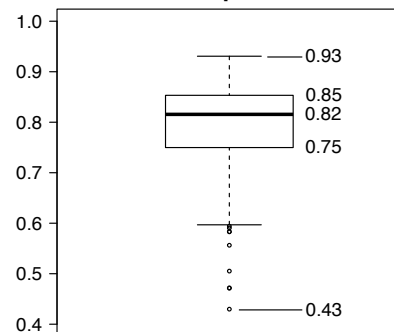**B****Observed**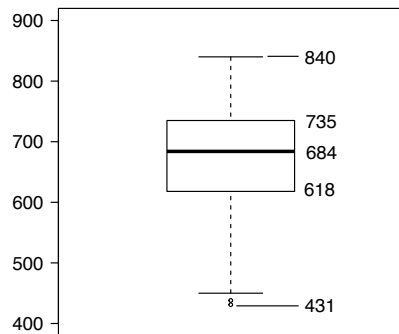**Shannon**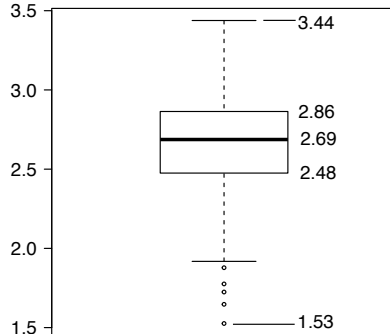**Simpson**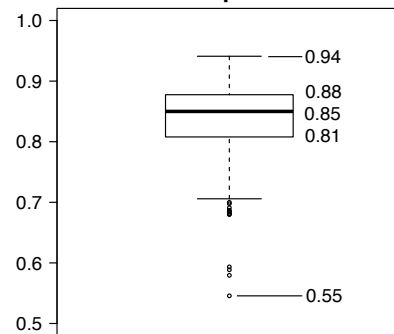

Supplement: Supplementary file 6 — Supplementary Figure 5 [file 41396_2022_1334_MOESM6_ESM.pdf]

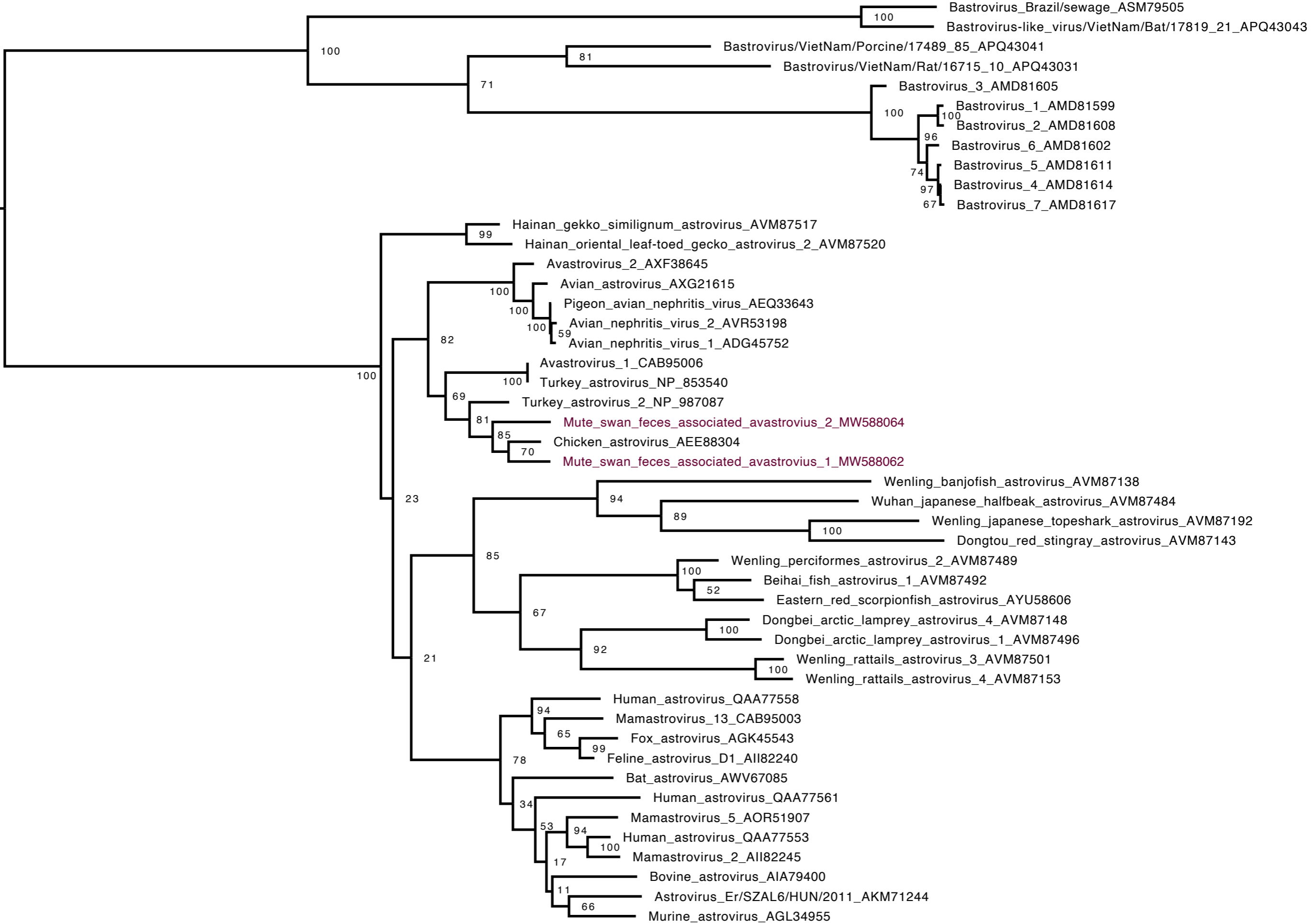

0.5

Supplement: Supplementary file 7 — Supplementary Figure 6 [file 41396_2022_1334_MOESM7_ESM.pdf]

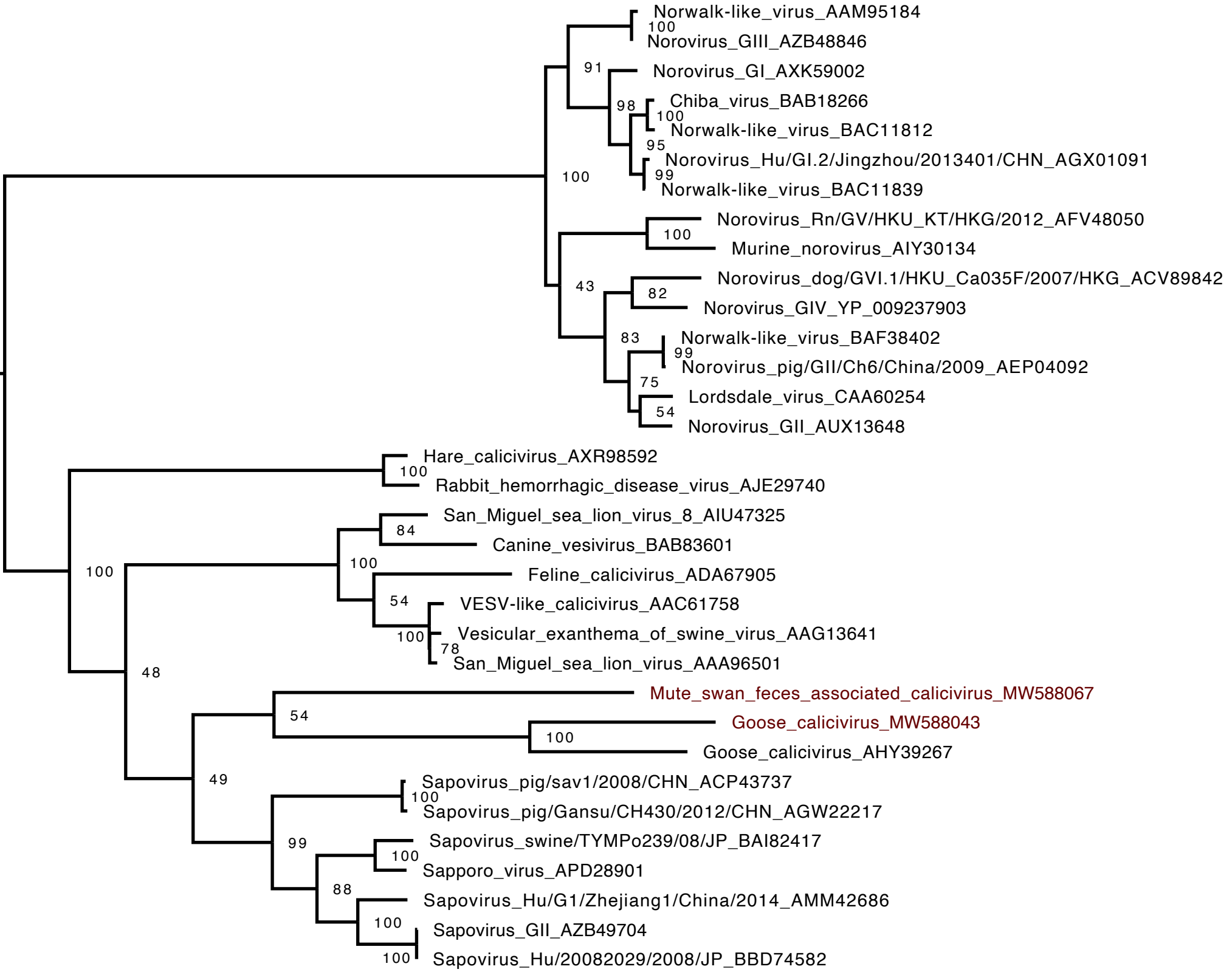

0.5

Supplement: Supplementary file 8 — Supplementary Figure 7 [file 41396_2022_1334_MOESM8_ESM.pdf]

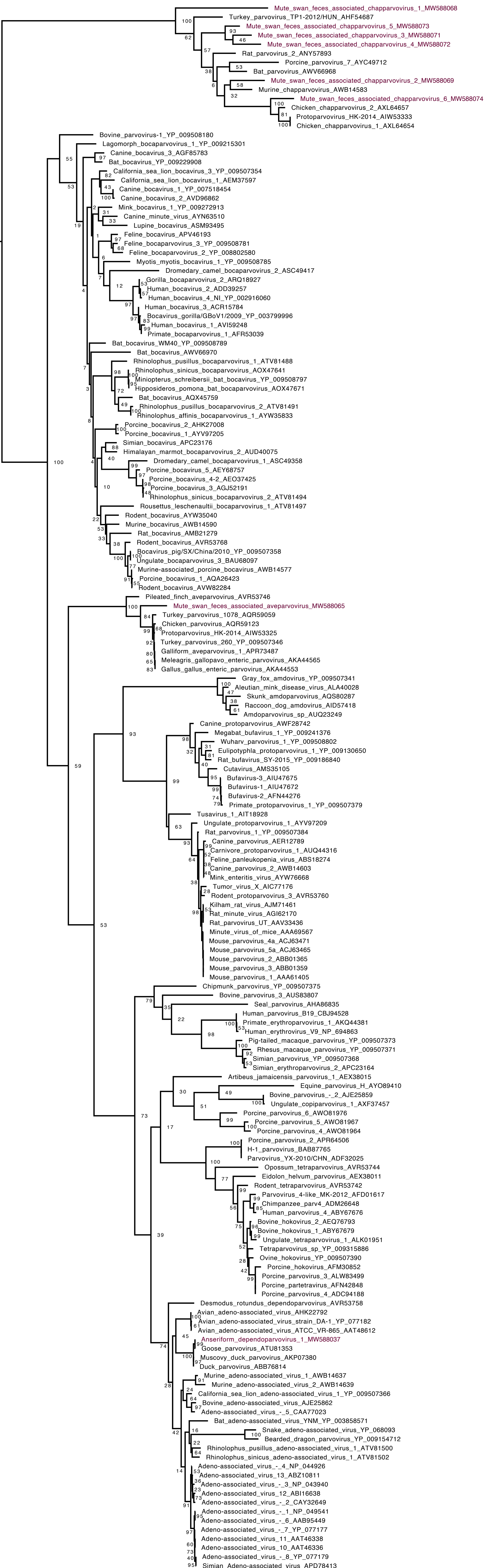

Supplement: Supplementary file 9 — Supplementary Figure 8 [file 41396_2022_1334_MOESM9_ESM.pdf]

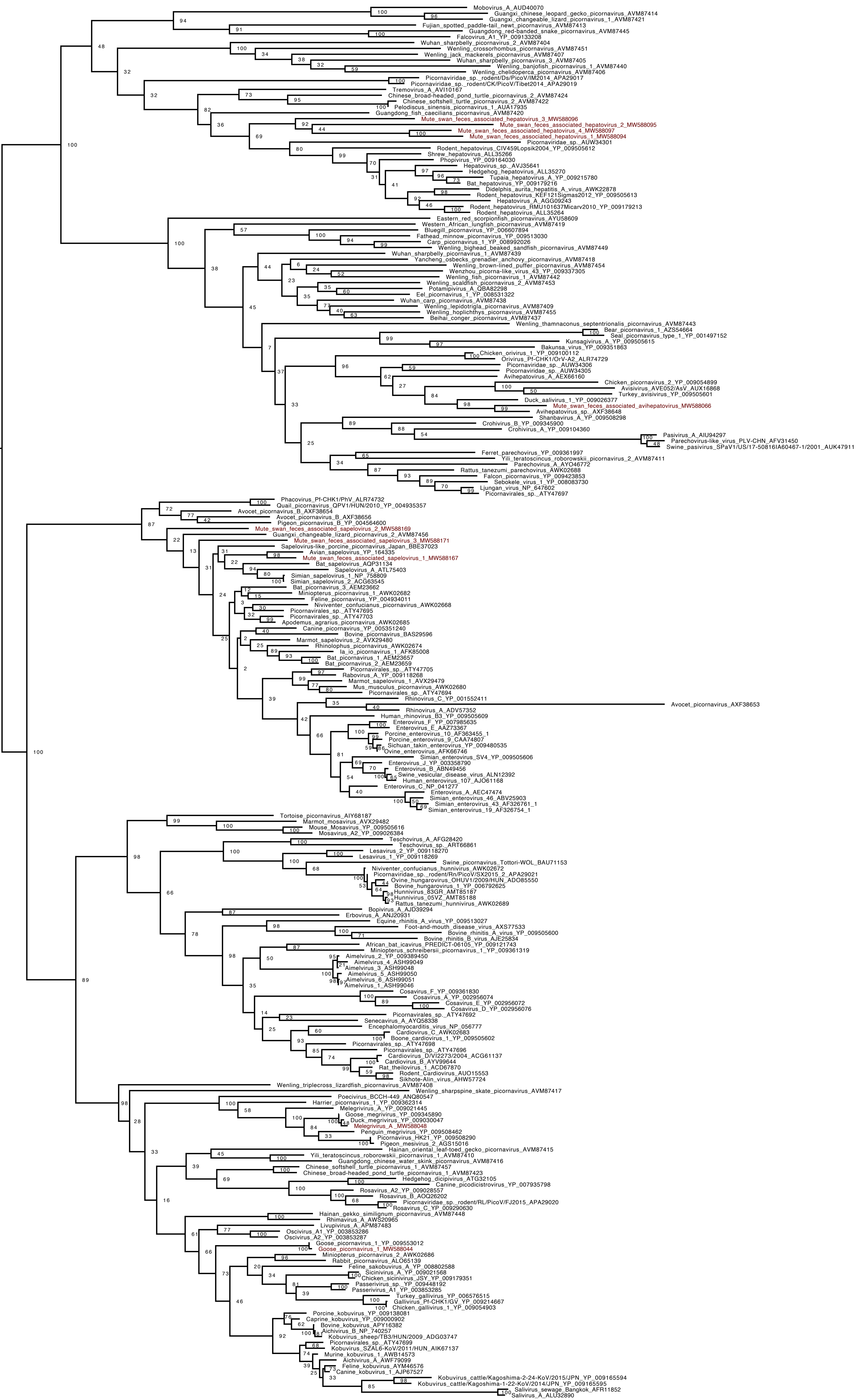

Supplement: Supplementary file 10 — Supplementary Figure 9 [file 41396_2022_1334_MOESM10_ESM.pdf]

(a) Picobirnaviridae – capsid protein

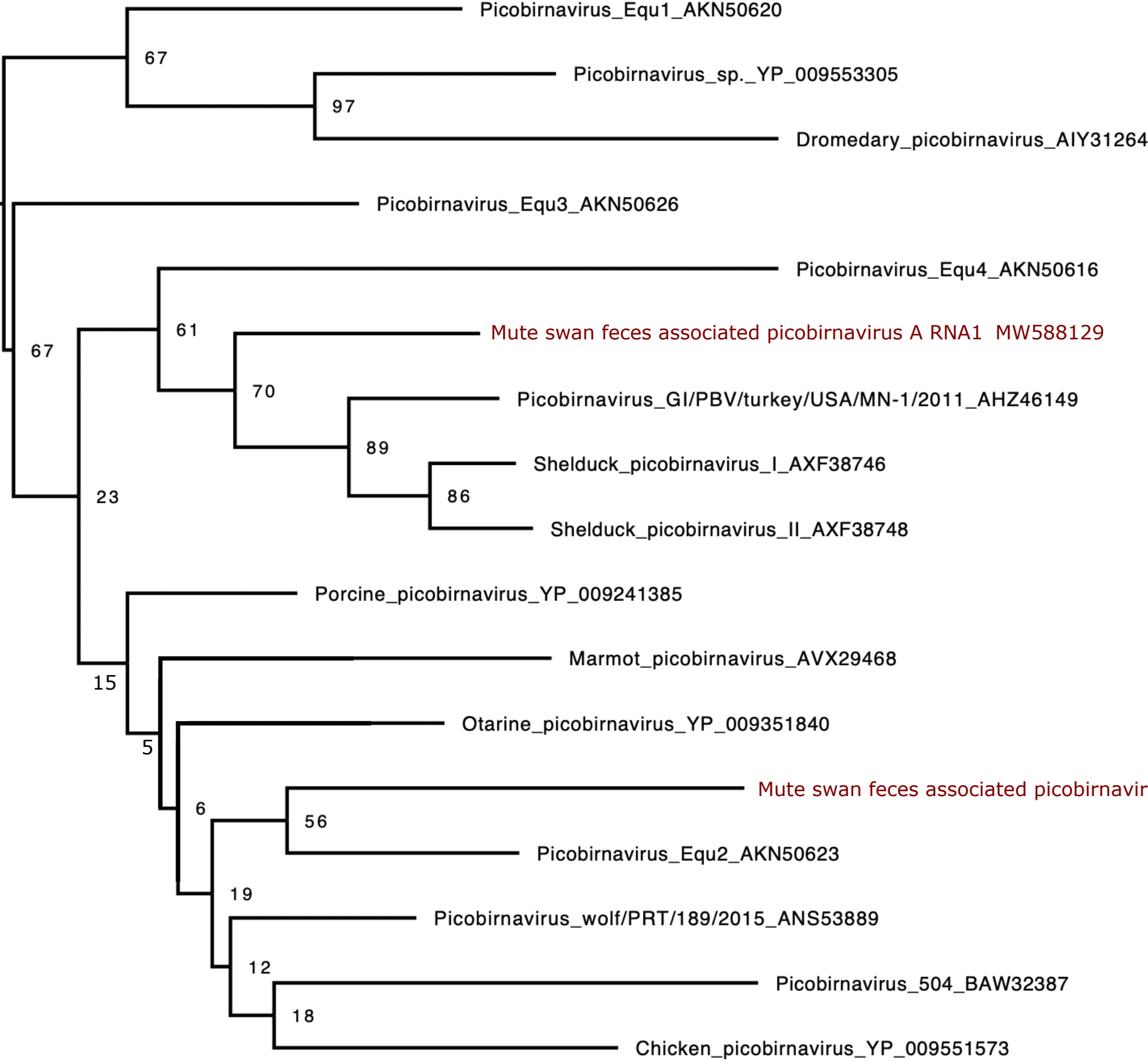

(b) Picobirnaviridae – RdRp protein

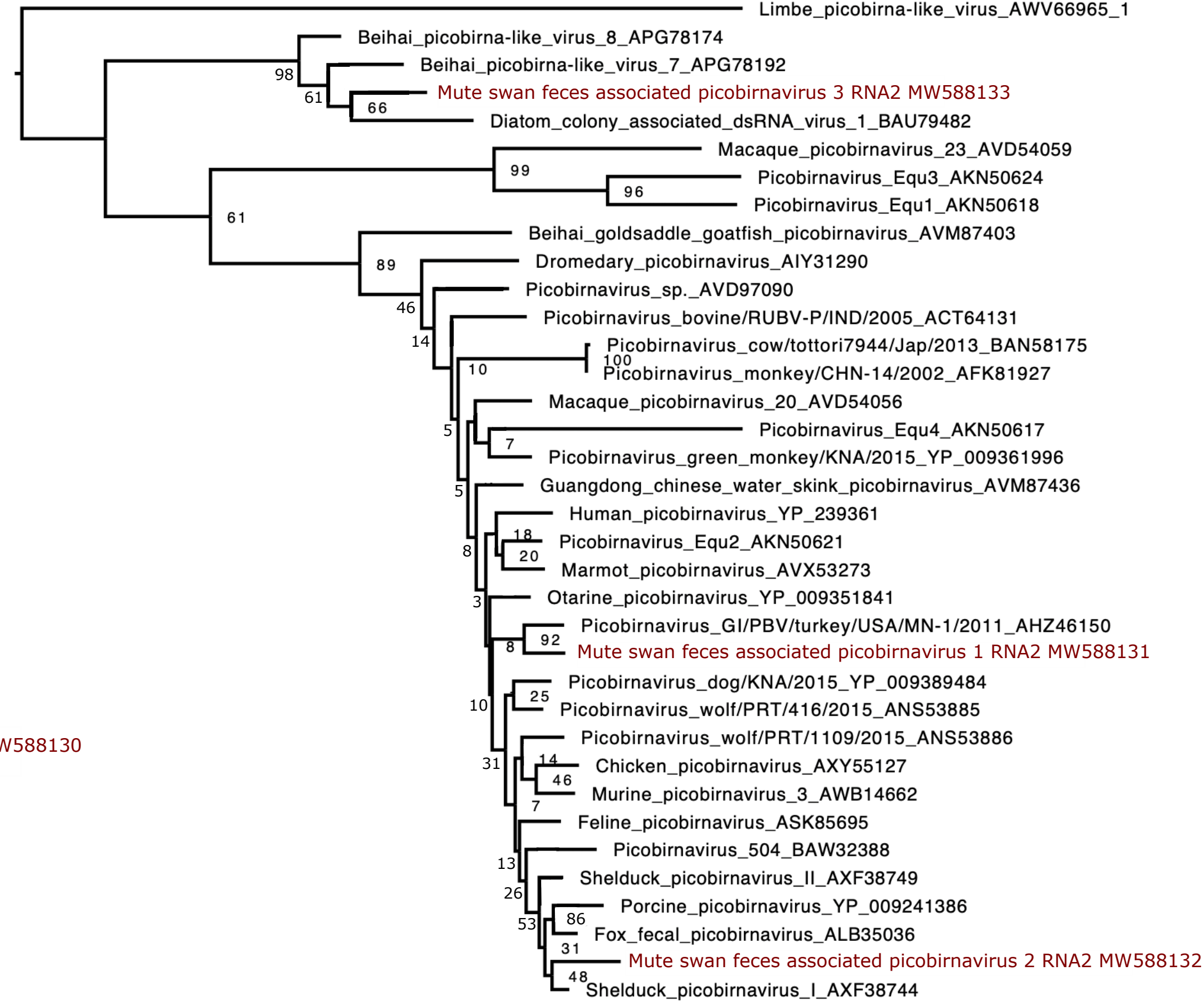

Supplement: Supplementary file 11 — Supplementary Figure 10 [file 41396_2022_1334_MOESM11_ESM.pdf]

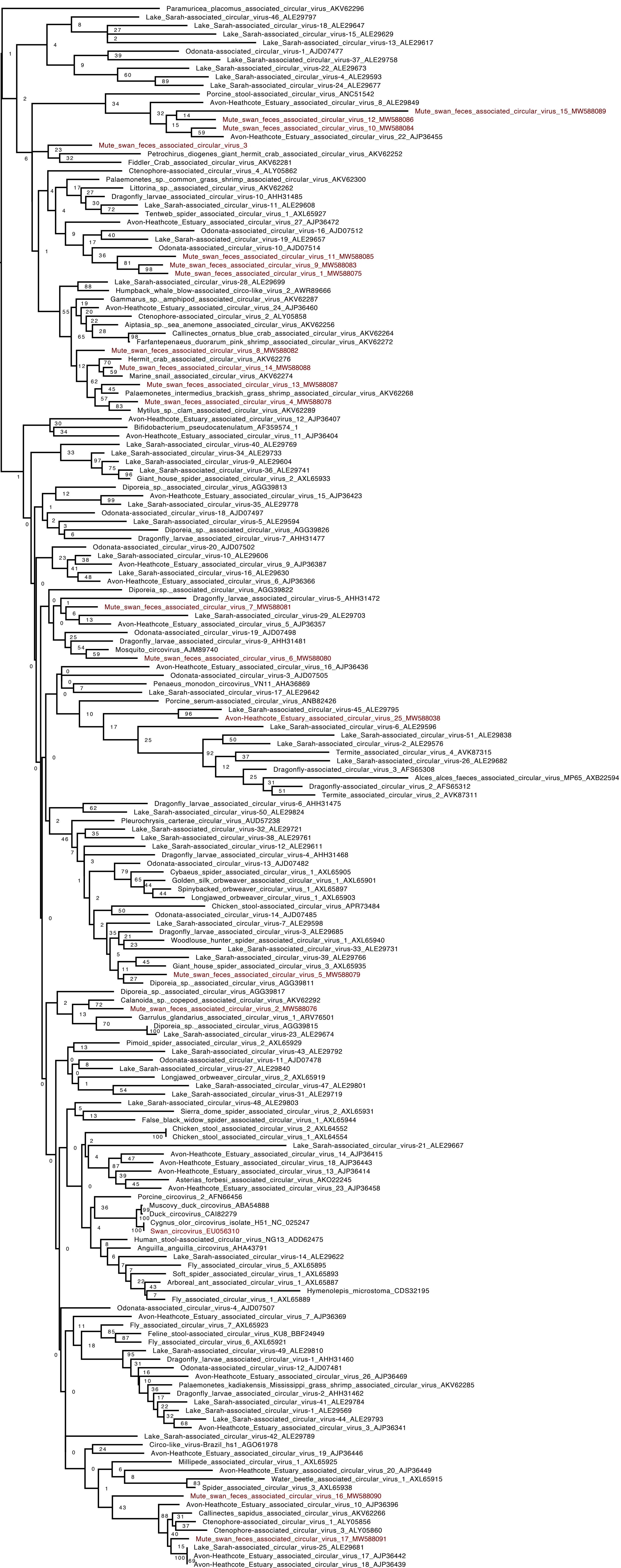

Supplement: Supplementary file 12 — Supplementary Figure 11 [file 41396_2022_1334_MOESM12_ESM.pdf]

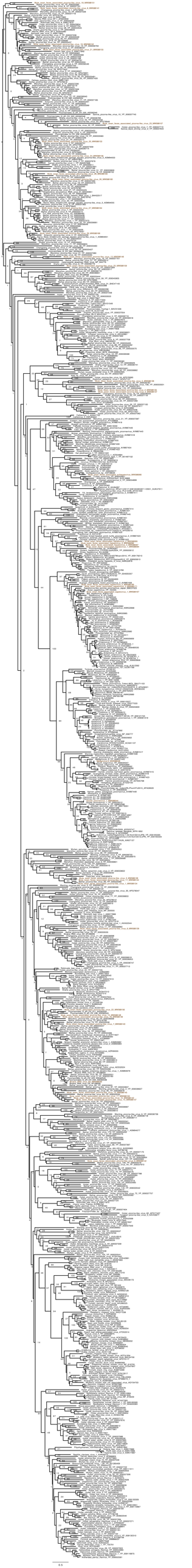

Supplement: Supplementary file 13 — Supplementary Figure 12 [file 41396_2022_1334_MOESM13_ESM.pdf]

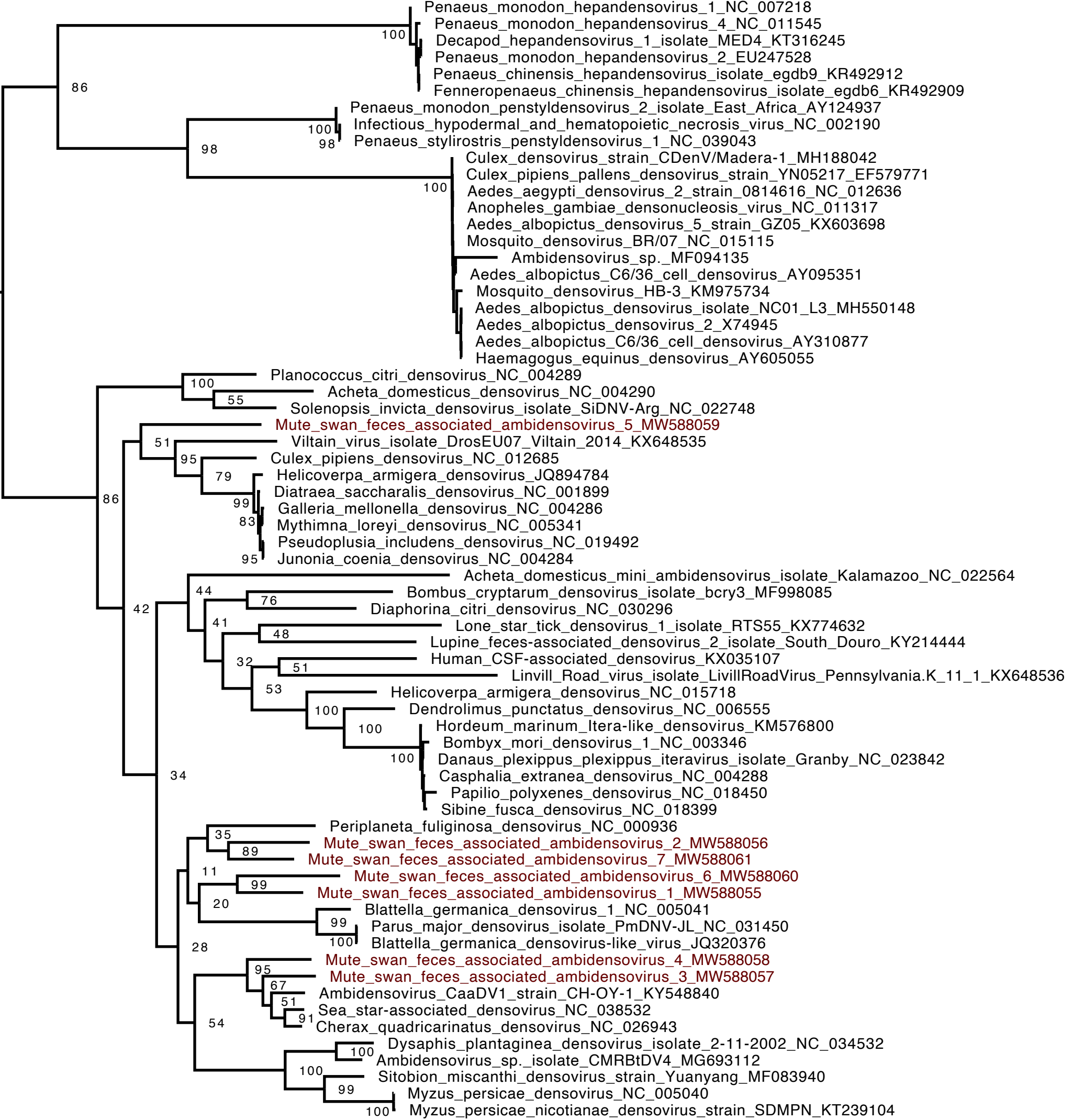

1.0

Supplement: Supplementary file 14 — Supplementary Figure 13 [file 41396_2022_1334_MOESM14_ESM.pdf]

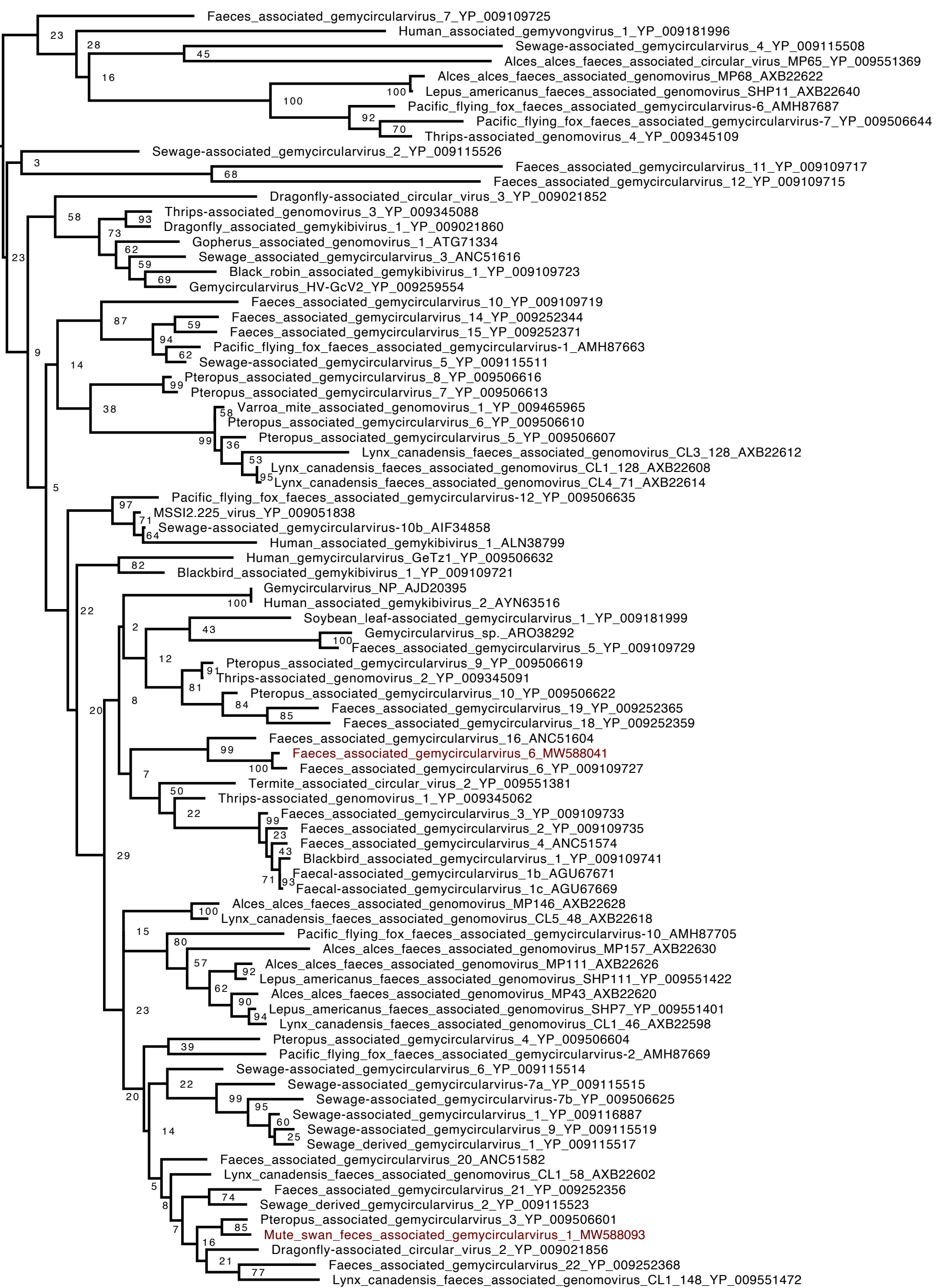

0.5

Supplement: Supplementary file 15 — Supplementary Figure 14 [file 41396_2022_1334_MOESM15_ESM.pdf]

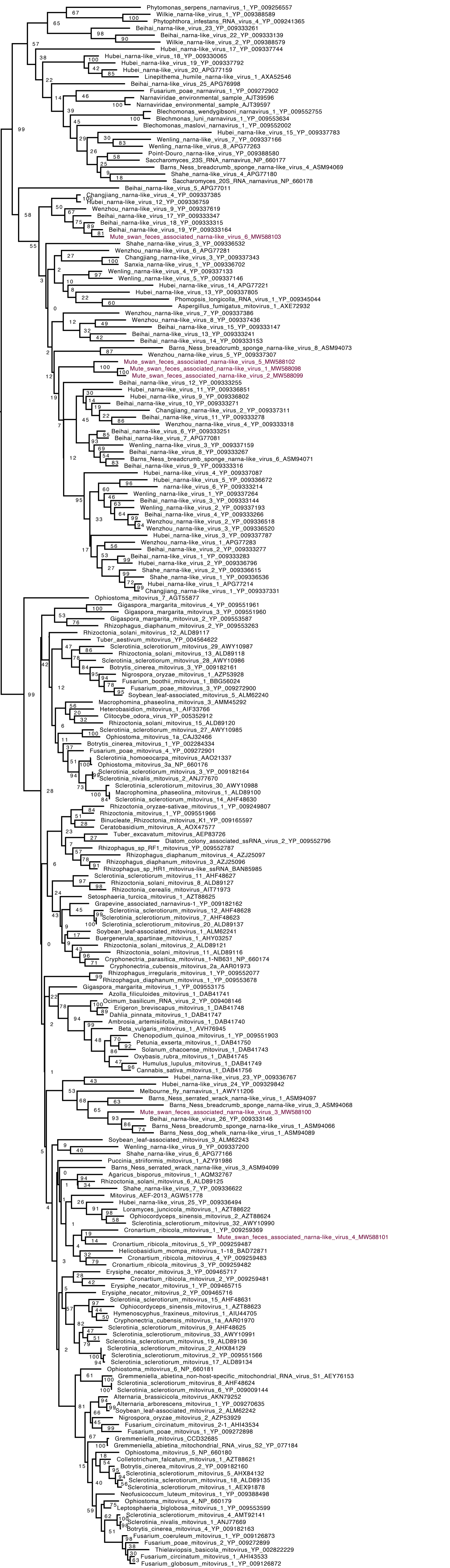

Supplement: Supplementary file 16 — Supplementary Figure 15 [file 41396_2022_1334_MOESM16_ESM.pdf]

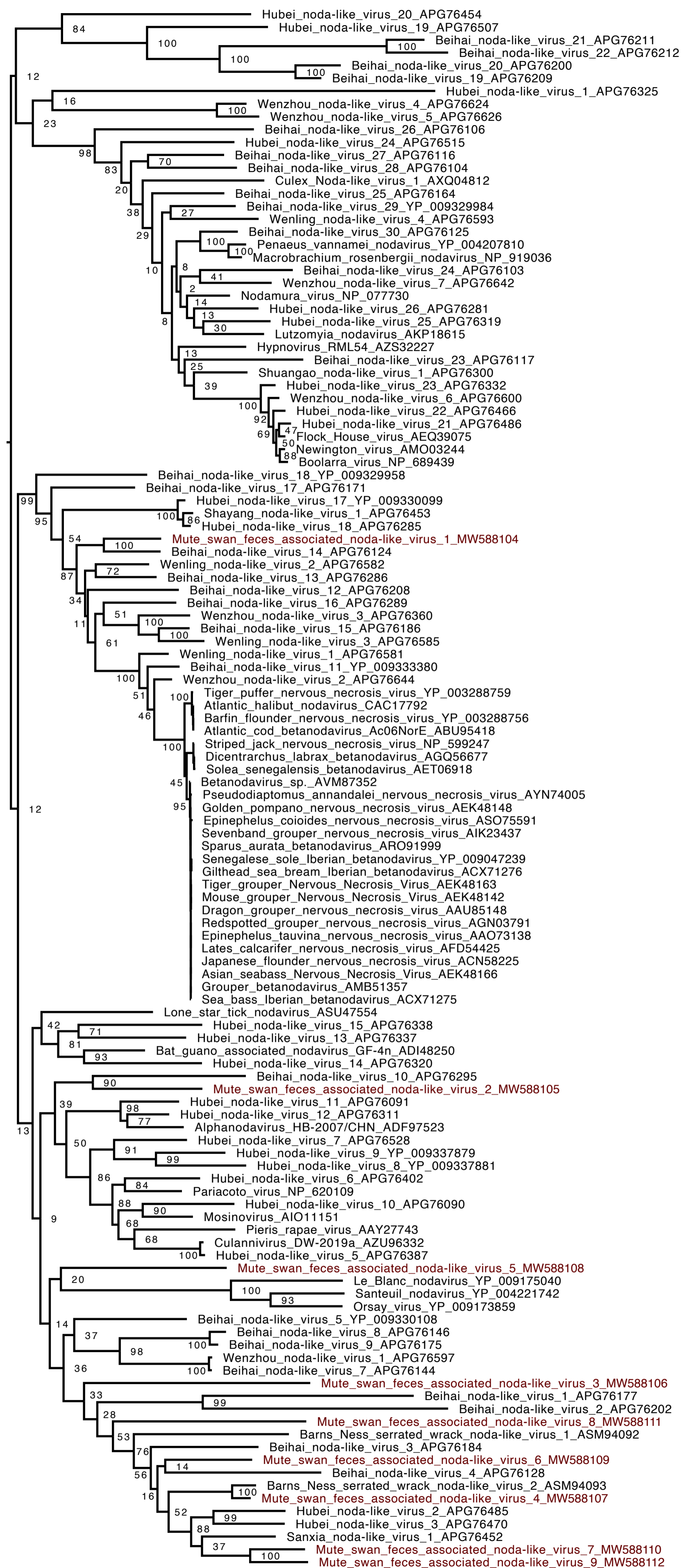

Supplement: Supplementary file 17 — Supplementary Figure 16 [file 41396_2022_1334_MOESM17_ESM.pdf]

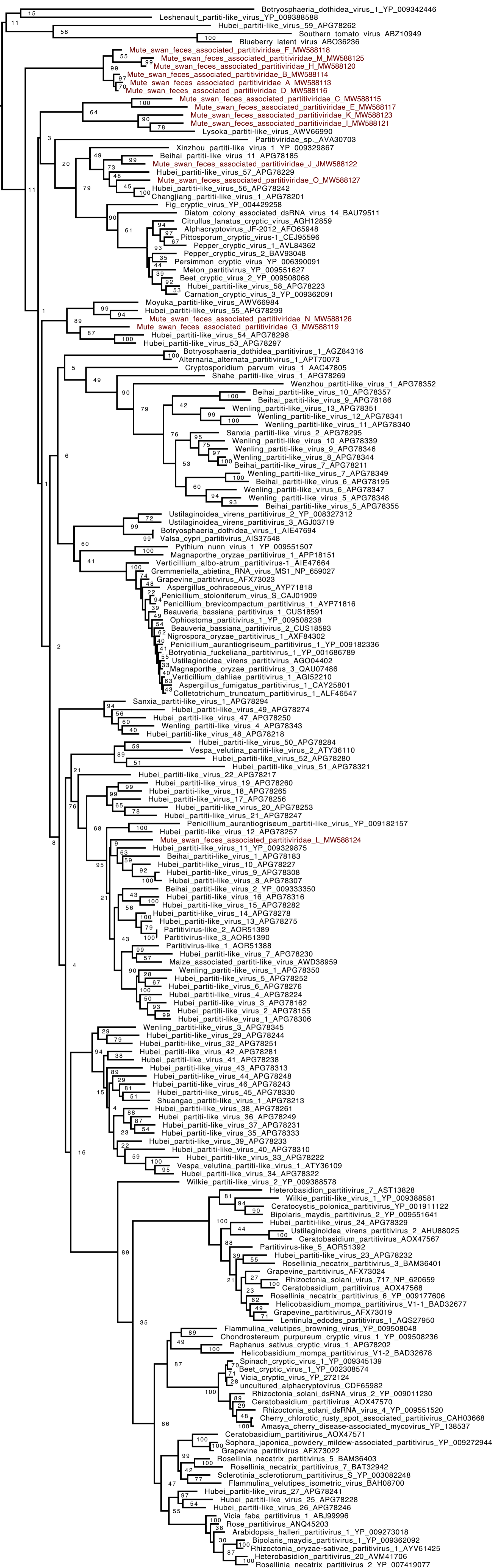

0.5

Supplement: Supplementary file 18 — Supplementary Figure 17 [file 41396_2022_1334_MOESM18_ESM.pdf]

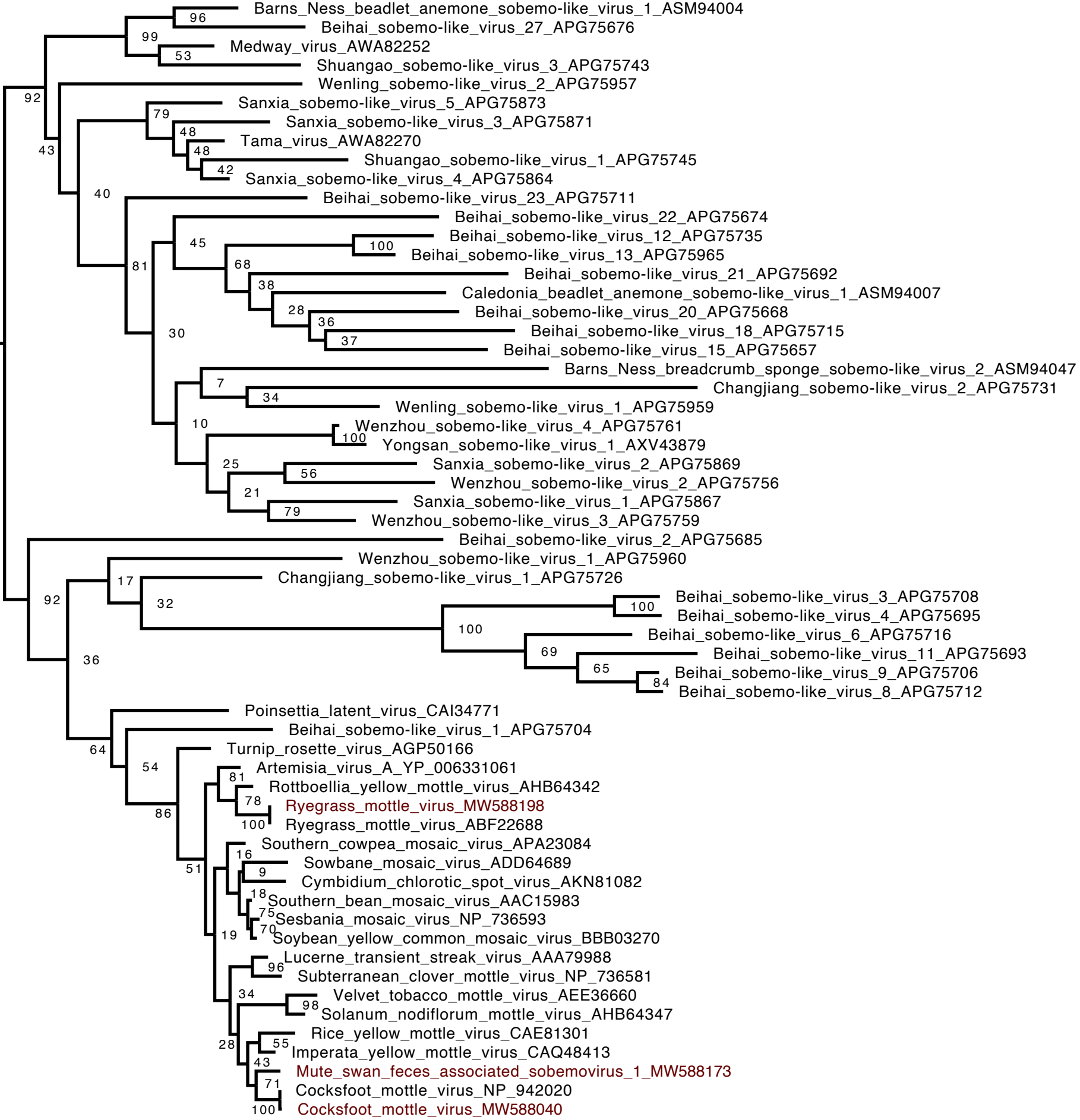

0.5

Supplement: Supplementary file 19 — Supplementary Figure 18 [file 41396_2022_1334_MOESM19_ESM.pdf]

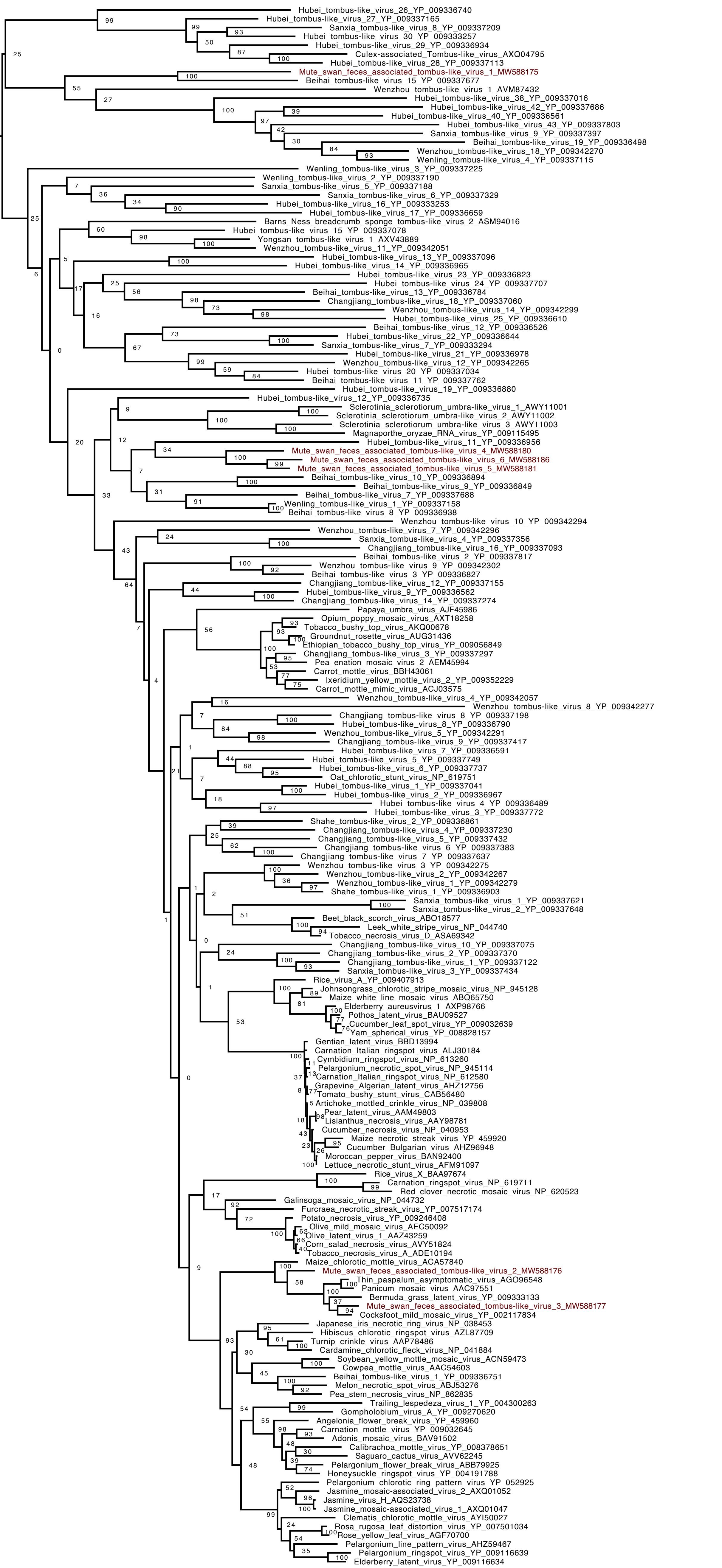

Supplement: Supplementary file 20 — Supplementary Figure 19 [file 41396_2022_1334_MOESM20_ESM.pdf]

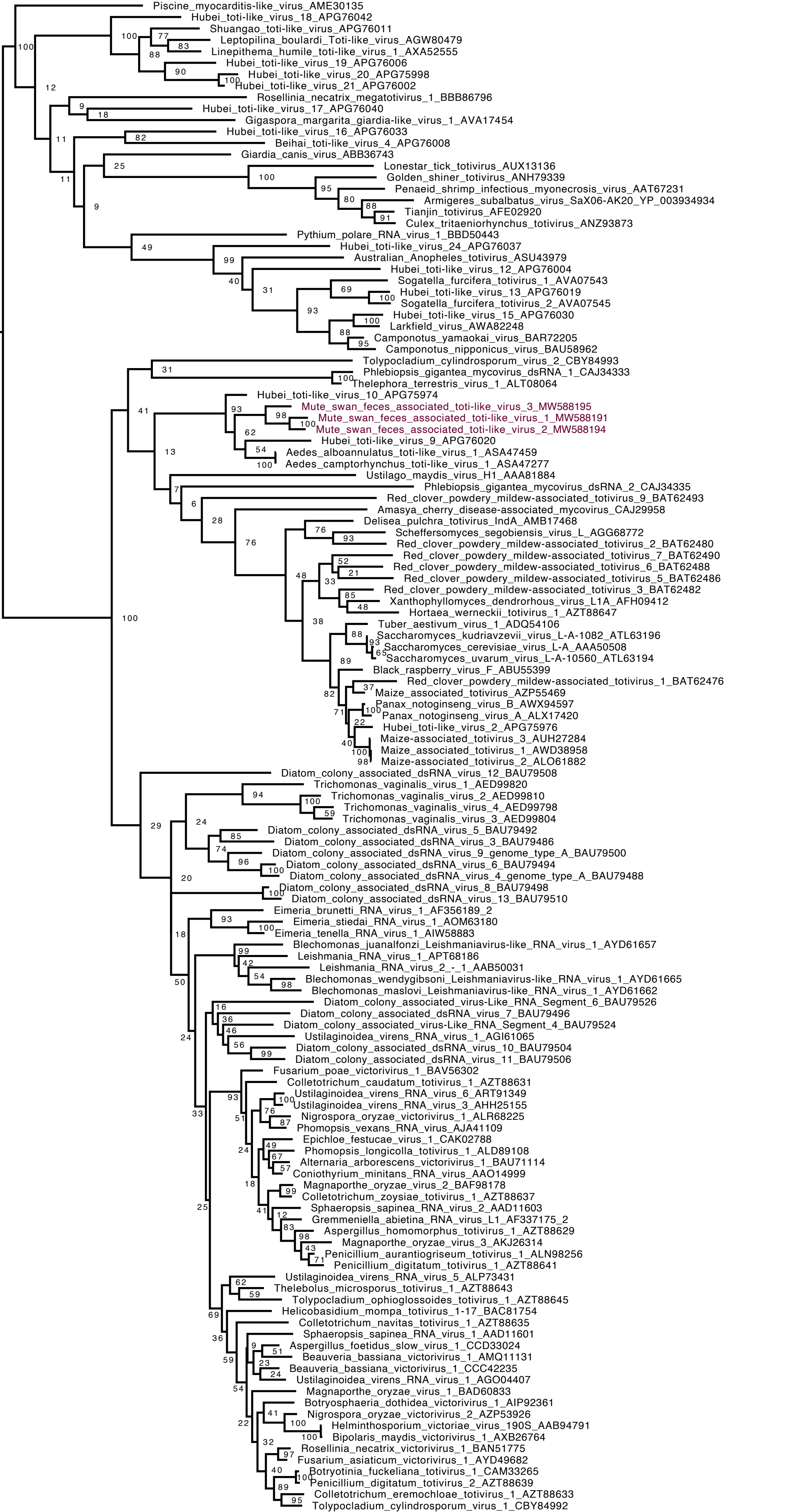

Supplement: Supplementary file 21 — Supplementary Figure 20 [file 41396_2022_1334_MOESM21_ESM.pdf]

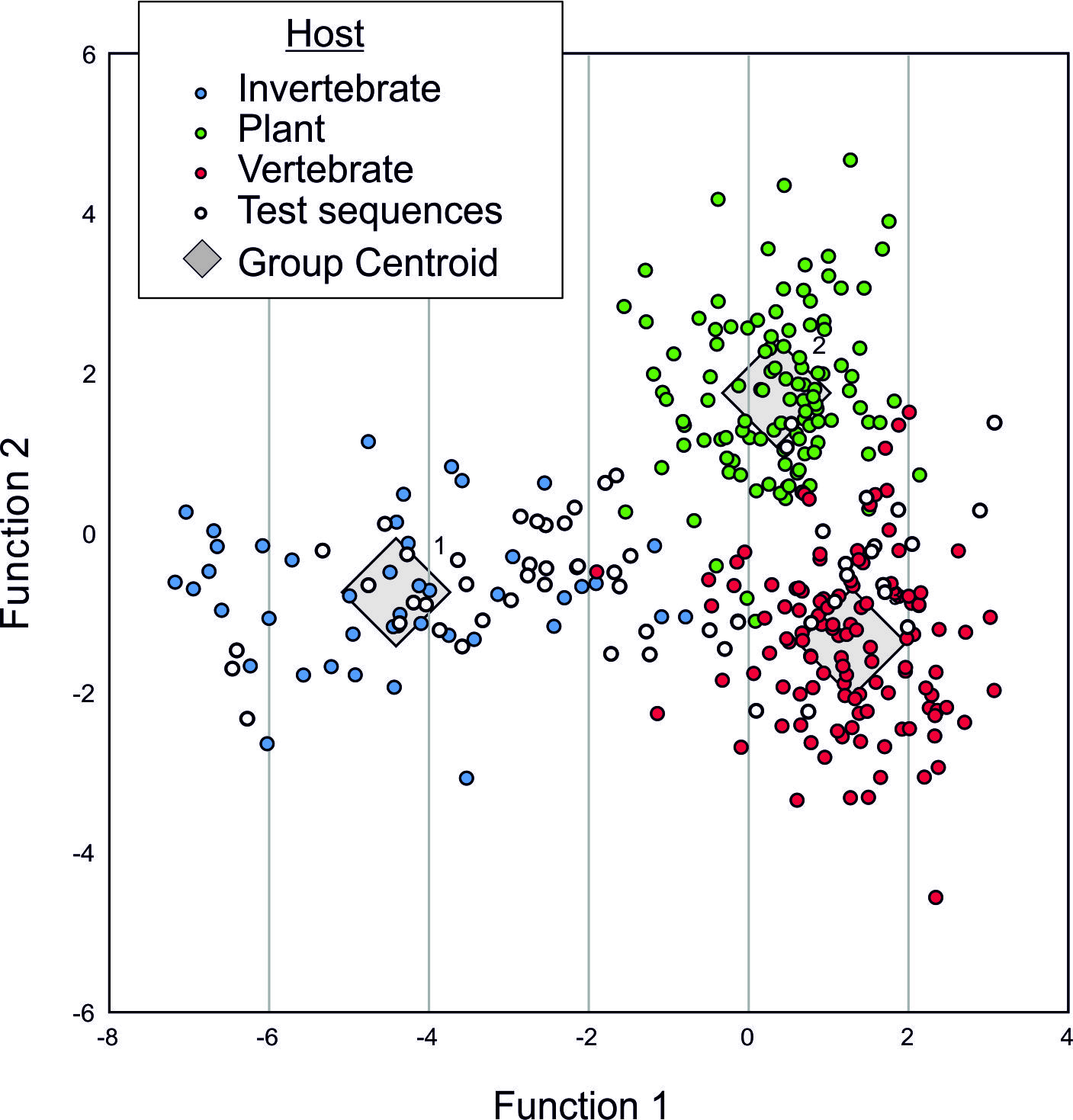

Supplement: Supplementary file 22 — Supplementary Figure 21 [file 41396_2022_1334_MOESM22_ESM.jpg]

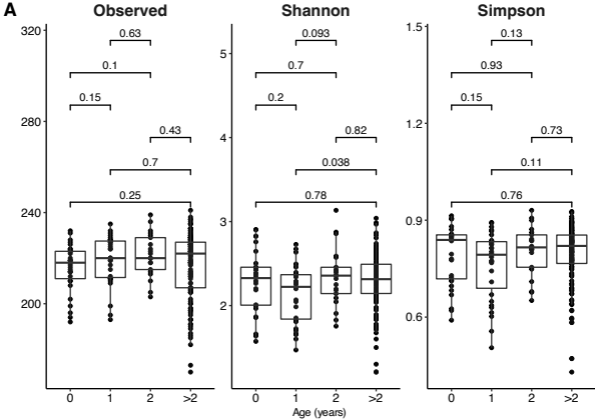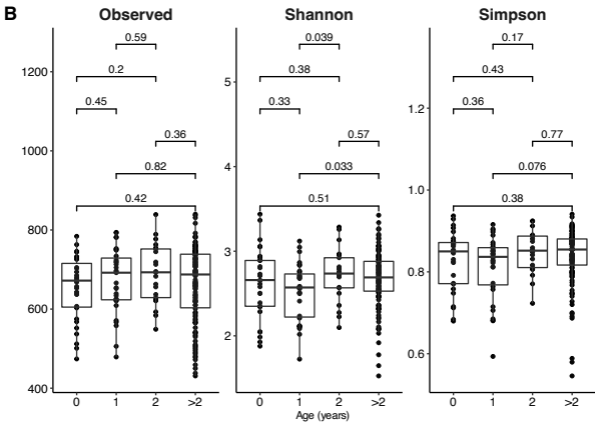

Supplement: Supplementary file 23 — Supplementary Figure 22 [file 41396_2022_1334_MOESM23_ESM.pdf]

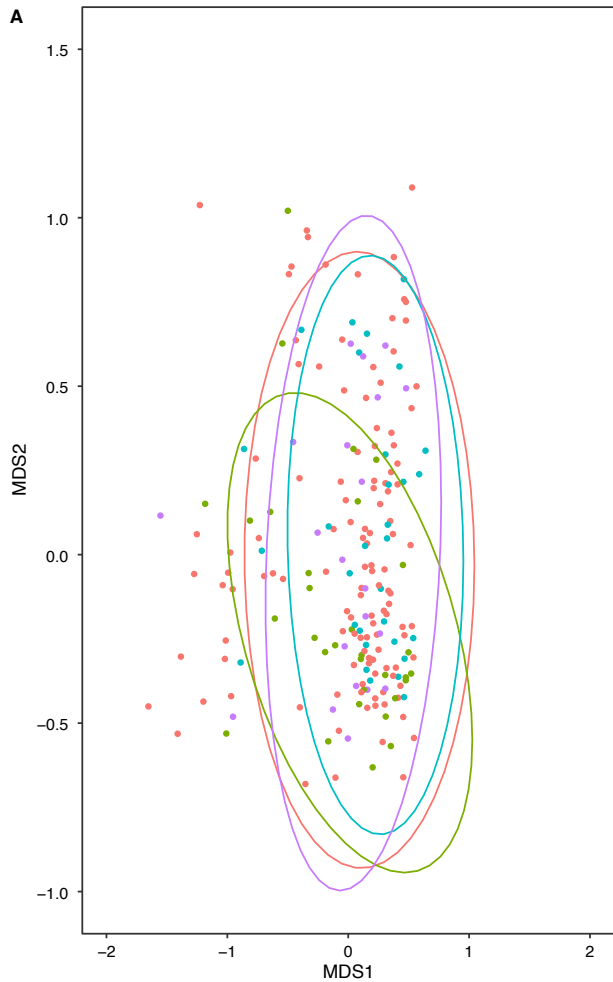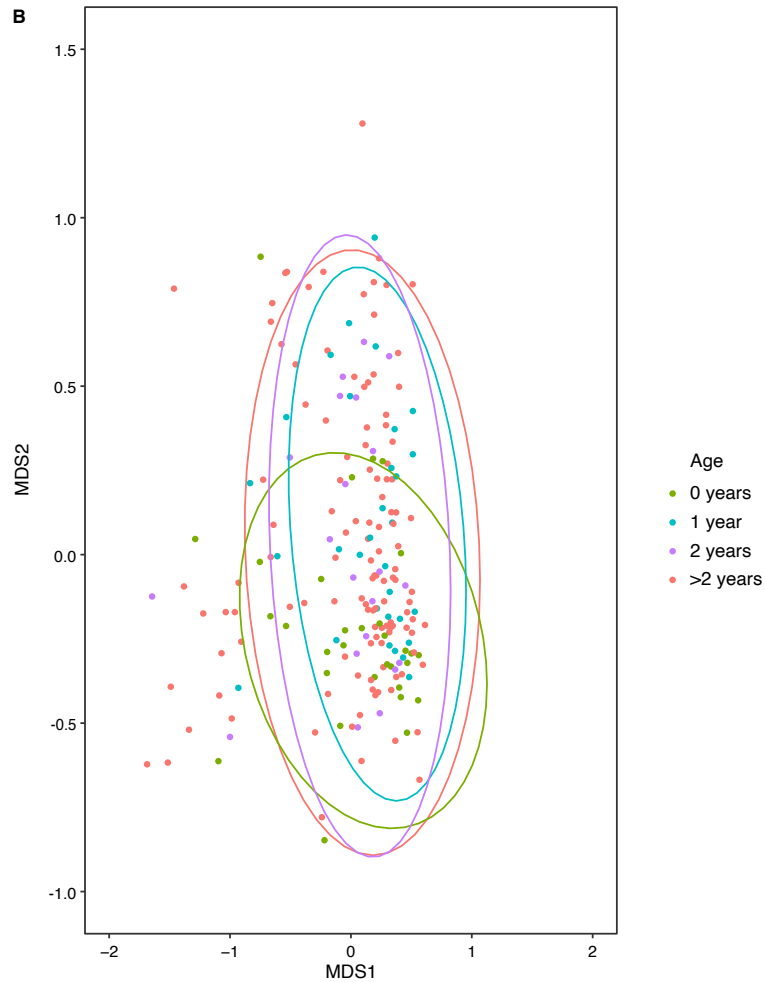

Supplement: Supplementary file 24 — Supplementary Figure 23 [file 41396_2022_1334_MOESM24_ESM.pdf]
